# Unraveling Unsupported Gold···Platinum Metallophilic Interactions: An In-Depth Computational and Topological Study

**DOI:** 10.1021/acs.inorgchem.5c01042

**Published:** 2025-06-25

**Authors:** Félix Reboiro, Daniel Blasco, M. Elena Olmos, José M. López-de-Luzuriaga, Miguel Monge

**Affiliations:** Departamento de Química, Instituto de Investigación en Química, Complejo Científico Tecnológico, 16764 Universidad de La Rioja, Madre de Dios 53, 26006 Logroño, La Rioja, Spain

## Abstract

We have computationally studied the attraction between gold and platinum atoms in a series of unsupported model systems with formulas *cis*/*trans*-[Pt­(CH_3_)_2_(NH_3_)_2_]­[Au­(CH_3_)_3_(NH_3_)] (**1a/1b**), *cis*/*trans*-[Pt­(CH_3_)_2_(NH_3_)_2_]­[Au­(CH_3_)­(NH_3_)] (**2a/2b**), [Pt­(CH_3_)­(NH_3_)_3_]­[Au­(CH_3_)_2_] (**3**), and {[Pt­(NH_3_)_4_]­[Au­(CH_3_)_2_]}^+^ (**4**). These systems stem from the simplification of the well-known orthometalated complexes of these metals, allowing for a clearer-as-possible description of the metallophilic interaction while reducing computation cost and keeping chemical representativeness. To achieve this goal, we fully optimized the model systems at the MP2 level of theory with the def2-TZVP basis sets. We analyzed the interaction energy at equilibrium distances through potential energy curves at the RHF and MP2 levels of theory, employing different relativistic pseudopotentials. Additionally, we examined various parameters, including natural bonding orbital effective charges or bond orders, and conducted an in-depth topological analysis of the electron density. The Au···Pt interaction has been characterized as a regular closed-shell interaction with some degree of electron sharing, but weaker (ca. 15 kJ·mol^–1^) than other metallophilic interactions, such as Au···Au or Au···Hg. Overall, this study sheds light on the key factors influencing the Au···Pt interaction.

## Graphical abstract

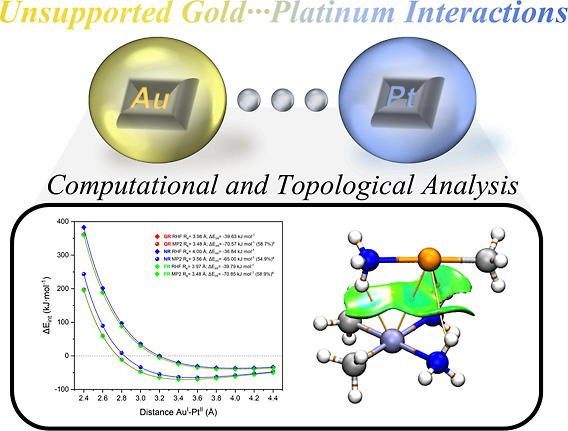

## 1 Introduction

Throughout the last three decades, the attractive interactions between gold­(I) and other closed-shell (d^10^, d^10^s^2^) or seemingly closed-shell (d^8^) metal systems, known as metallophilicity,^1,2^ have received significant attention from both experimental and theoretical viewpoints.^3–6^ Extensive research has focused on unraveling the nature of these weak noncovalent interactions. Far from being innocent, these interactions play a significant role in stabilizing reactive intermediates in catalysis,^7–9^ driving supramolecular assemblies,^10,11^ vapochromism,^12^ mechanochromism,^13^ sensing,^14^ and luminescence,^15^ particularly in materials for OLEDs.^16^

Metallophilic interactions refer to the counterintuitive attraction between two closed-shell metal cations, including homometallophilic interactions of Au^I^···Au^I^,^17,18^ Au^III^···Au^III^,^19,20^ Pt^II^···Pt^II^,^21,22^ Ag^I^···Ag^I^,^23^ and Hg^II^···Hg^II^,^24^ and heterometallophilic interactions such as Au^I^···Ag^I^,^25^ Au^I^···Hg^II^,^26^ and Pt^II^···Ag^I^,^27^ among them. These are weak noncovalent forces of van der Waals type (ranging from 20 to 50 kJ·mol^–1^, similar to hydrogen bonding interactions in strength),^28^ arising from dispersion-type correlation effects that are further strengthened by the large relativistic effects of heavy atoms.^5,29,30^ Additionally, when the distance between closed-shell metals is shorter than their respective sum of van der Waals radii, it is generally considered an indication of metallophilicity. However, since different radii are proposed for the same atom,^31–33^ this criterion is not a definitive proof. Despite considerable computational efforts at density functional theory (DFT) and post-Hartree–Fock (HF) levels of theory that consider the electron correlation effect, such as MP2, SCS-MP2 or CCSD­(T), their nature remains controversial and there is no consensus in the scientific community.^34^

Gold-containing heterometallic compounds are of great interest due to their tunable metallophilic interactions.^35–37^ Homometallic systems featuring Au^I^···Au^I^ and Pt^II^···Pt^II^ interactions, which constitute a recurring structural motif, are well-documented in the literature.^3–6,15^ However, comparatively fewer examples of Au···Pt interactions have been reported. This is, in principle, unexpected because the linear or square planar geometry of gold­(I) and gold­(III), respectively, and the square planar geometry of platinum­(II), along with the significant relativistic effects of both atoms, create a suitable model for the formation of such interactions.

One of the structural frameworks of dinuclear gold–platinum heterometallic complexes involves the use of two bridging ligands that bring the metal centers into proximity, see Scheme 1, **1A**. In this context, Che et al. reported the spectroscopic properties and X-ray structures of heterodinuclear complexes with two dcpm (1,2-bis­(dicyclohexylphosphino)­methane) ligands.^38^ The complex [Pt­(CN)_2_(μ-dcpm)_2_Au] (Scheme 1, **1B**) revealed short Au^I^–Pt^II^ distances and a red shift in the absorption bands. Recently, Gericke et al. published a synthetic approach for bis­(chelate) complexes of the type *trans*-[M­(κ^2^-2-C_6_F_4_PPh_2_)_2_] (M = Ni, Pt) and *cis*-[Pt­(κ^2^-2-C_6_F_4_PPh_2_)_2_], with monovalent coinage metal ions.^39^ They synthesized several heterobimetallic complexes displaying supported Au^I^···Pt^II^ interactions (Scheme 1, **1C**), whose presence was supported by computational and topological analysis.

**Scheme 1:**
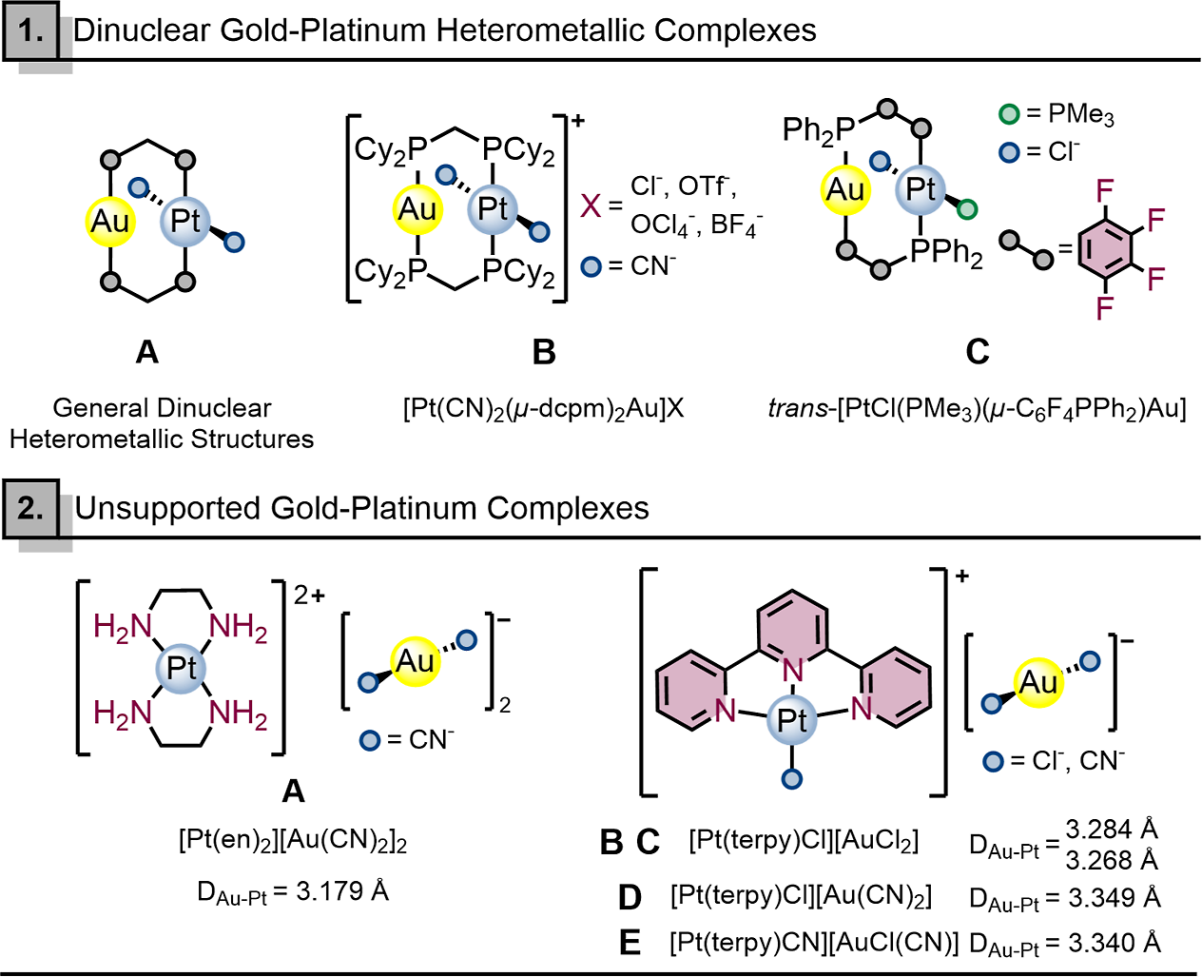
Representative Examples of Dinuclear and Unsupported Gold–Platinum Complexes Reported in the Literature, Illustrated by Compounds **1A–1C** and **2A–2E**, Respectively

Nevertheless, very little is known about unsupported gold–platinum interactions, with a surprisingly low number of complexes of this type reported. In them, the metallophilic interactions occur between oppositely charged ions with the Coulombic attraction appearing to be the key factor. In this regard, Balch et al. synthesized a series of double Ag^I^···Pt^II^ and Pt^II^···Au^I^ salts.^40^ Specifically, the crystal structure of the complex [Pt^II^(en)_2_]­[Au^I^(CN)_2_]_2_ (en = ethylenediamine) exhibits a linear chain-like packing of alternating cations and anions, with Au^I^–Pt^II^ distances of 3.1799(3) Å, see Scheme 1, **2A**. Moreover, Doerrer et al. synthesized, through metathesis reactions in an aqueous solution, nine double salts containing [Au^III^(bipy)­X_2_]^+^ or [Pt^II^(terpy)­X]^+^ cations (bipy = 2,2′-bipyridine; terpy = 2,2′:6′,2″-terpyridine; X = Cl, Br, CN), and [AuBr_4_]^−^, [AuCl_2_]^−^, or [Au­(CN)_2_]^−^ anions.^41^ Their recrystallization led to four infinite chains, of formula {[Pt­(terpy)­Cl]_2_[AuCl_2_]}­[AuCl_4_]·2DMF (DMF = *N*,*N*-dimethylformamide), {[Pt­(terpy)­Cl]_2_[AuCl_2_]}­[AuCl_4_]·CH_3_CN, [Pt­(terpy)­Cl]­[Au­(CN)_2_], and [Pt­(terpy)­CN]­[AuCl­(CN)], bearing Au^I^···Pt^II^ interactions with distances shorter than the sum of their van der Waals radii see Scheme 1, **2B–2E**, respectively.

On the other hand, our research group has used the acid–base strategy, in which a Lewis acid metal salt reacts with a basic gold complex, such as the well-known bis­(perhalophenyl)­aurate­(I) anion [AuR_2_]^−^ (R = C_6_F_5_, C_6_Cl_5_ and C_6_F_3_Cl_2_), allowing us to isolate heterometallic complexes bearing unsupported Au^I^···M interactions (M = Ag,^42,43^ Pb,^44–46^ Hg,^47^ Tl,^48,49^ and Bi^50^). In view of our previous experimental and theoretical results,^51^ we report a full computational and topological study at the correlated MP2 level of theory on simplified model systems displaying unsupported Au···Pt interactions. This investigation enables us to understand the nature and strength of these interactions, predicting their feasibility in future experimental work.

## 2 Computational Details

All calculations were performed using the Gaussian 16 suite of programs^52^ at the RHF,^53^ MP2,^54,55^ and DFT-PBE0 ^56–58^ levels of theory. The Karlsruhe def2-TZVP basis sets^59^ were employed for all atoms; 2f-type polarization functions and a pseudorelativistic 60-electron effective core potential (def2-ECP)^60^ were used for gold and platinum. Additionally, we conducted calculations with both nonrelativistic (ECP60MHF)^61^ and fully relativistic (FR) (ECP60MDF)^62,63^ 60-electron effective core potentials (ECP).

Full optimizations were carried out at the RHF/def2-TZVP and MP2/def2-TZVP levels of theory, and frequency analyses at the same level of theory confirmed that the geometries correspond to minima (no imaginary frequencies were found). The structures were visualized and rendered using GaussView^64^ and UCSF ChimeraX programs.^65^

The interaction energies (Δ*E*_int_) were obtained at the MP2 and RHF levels of theory with the counterpoise correction (cp) to the basis set superposition error (BSSE),^66^ see eq S1. The calculated points were then fitted using the Herschbach–Laurie four-parameter function^67^ to represent the potential energy curves (PECs), see eq S2. To refine the interaction energies of the minima, single-point counterpoise-corrected calculations were performed at the SCS-MP2 ^68^ and DLPNO-CCSD­(T)^69^ levels of theory using the def2-TZVP basis set. The RIJCOSX^70^ approximation and the corresponding default auxiliary basis sets for def2-TZVP were employed as implemented in the ORCA 5 package.^71^

Natural bonding orbital (NBO)^72^ and Wiberg bond index (WBI)^73^ calculations were performed using the Gaussian 16 package. The intrinsic bond strength index (IBSI),^74^ delocalization index (DI),^75^ and quantum theory of atoms in molecules (QTAIM) charges^76^ were computed with Multiwfn software.^77^ These calculations were performed at the MP2/def2-TZVP level of theory.

Natural energy decomposition analysis (NEDA)^78,79^ was carried out using Gaussian 16 and NBO 7.0 programs^80^ at the hybrid functional PBE0 (Gaussian keyword PBE1PBE) using the def2-TZVP basis sets with the third empirical dispersion correction by Grimme D3­(BJ).^81,82^

On the other hand, we have also performed a topological analysis of the MP2/def2-TZVP electron density using QTAIM,^76^ interaction region indicator (IRI),^83^ and independent gradient model based on Hirshfeld partition (IGMH)^84^ methodologies computed with Multiwfn software. The representations of the electron density studied were visualized and analyzed with the VMD program.^85^ Additional computational details and equations are provided in the Supporting Information.

## 3 Results and Discussion

### 3.1 Geometries and Optimization of the Models

Computational models have been designed with the purpose of isolating the gold···platinum interactions in neutral and cationic complexes. They stem from the simplification of the well-known orthometalated complexes,^86,87^ by substituting the donor groups with methyl and ammonia ligands for a clearer-as-possible description of the metallophilic interaction without sacrificing chemical representativeness. Our group previously used successfully a similar approach to study Au^III^···Au^III^ interactions.^51^

Thus, neutral models with the formula *cis*-[Pt­(CH_3_)_2_(NH_3_)_2_]­[Au­(CH_3_)_3_(NH_3_)] (model **1a**) and *trans*-[Pt­(CH_3_)_2_(NH_3_)_2_]­[Au­(CH_3_)_3_(NH_3_)] (model **1b**) were built for the study of the Au^III^···Pt^II^ interaction. Neutral models with formula *cis*-[Pt­(CH_3_)_2_(NH_3_)_2_]­[Au­(CH_3_)­(NH_3_)] (model **2a**), *trans*-[Pt­(CH_3_)_2_(NH_3_)_2_]­[Au­(CH_3_)­(NH_3_)] (model **2b** and **2c**), and [Pt­(CH_3_)­(NH_3_)_3_]­[Au­(CH_3_)_2_] (model **3**) were constructed for studying the Au^I^···Pt^II^ interaction. Finally, model **4** with formula {[Pt­(NH_3_)_4_]­[Au­(CH_3_)_2_]}^+^ was designed to explore the latter interaction in cationic complexes, see Scheme S1.

All molecular structures were optimized at the MP2/def2-TZVP level of theory without geometry constraints, except for model **2c**, where the intermetallic distance was kept fixed (more details in the Supporting Information). The linear and square planar geometries for the Au^I^ and Au^III^, Pt^II^ fragments enable the interaction between the metal centers, positioned in almost parallel planes, see Figure 1. Additionally, a selection of bond lengths and angles of the optimization structures are listed in Table S1. Through the intermetallic distance, a qualitative assessment of the attraction between the metal centers can be evaluated. Hence, intermetallic distances close to or shorter than the sum of the van der Waals radii of the metals (i.e. 3.41 Å or 4.61 Å)^31–33^ suggest a potential attractive interaction; however, this alone does not provide definitive proof of it.

**Figure 1:**
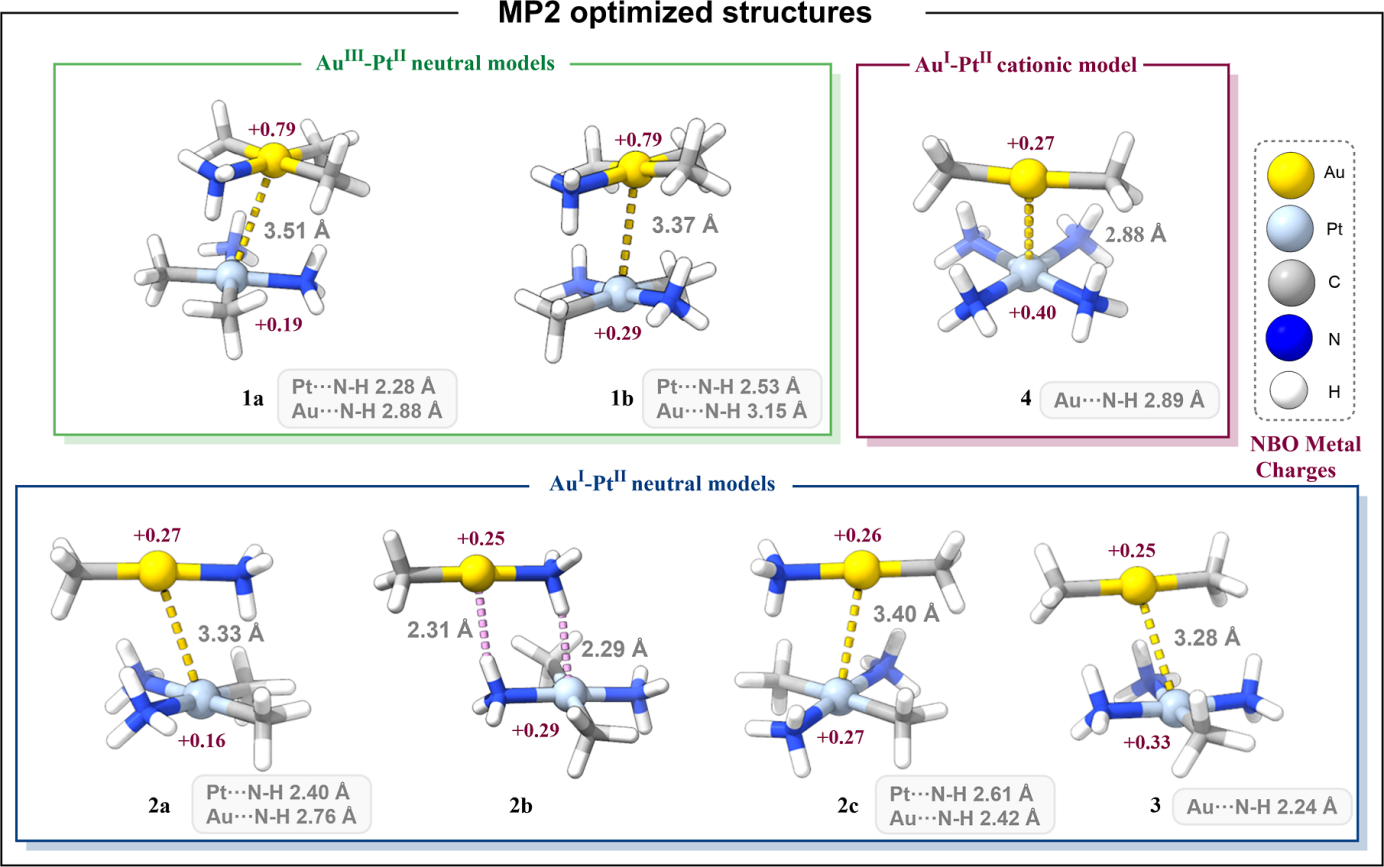
Representations of the MP2/def2-TZVP optimized structures of models **1–4**. The most relevant distances and the NBO metal charges of the optimization are included.

When compared with the structures optimized at the RHF/def2-TZVP level of theory (see Figure 2), it is clear that the inclusion of the electron correlation is crucial. Dispersion-type interactions, which play a key role in stabilizing metallophilic systems, are inherently captured through electron correlation. Nevertheless, it should be emphasized that while all dispersion arises from correlation effects, not all correlation can be attributed to dispersion. This distinction is particularly relevant when evaluating the contributions to the interaction energy using correlated methods such as MP2.

**Figure 2:**
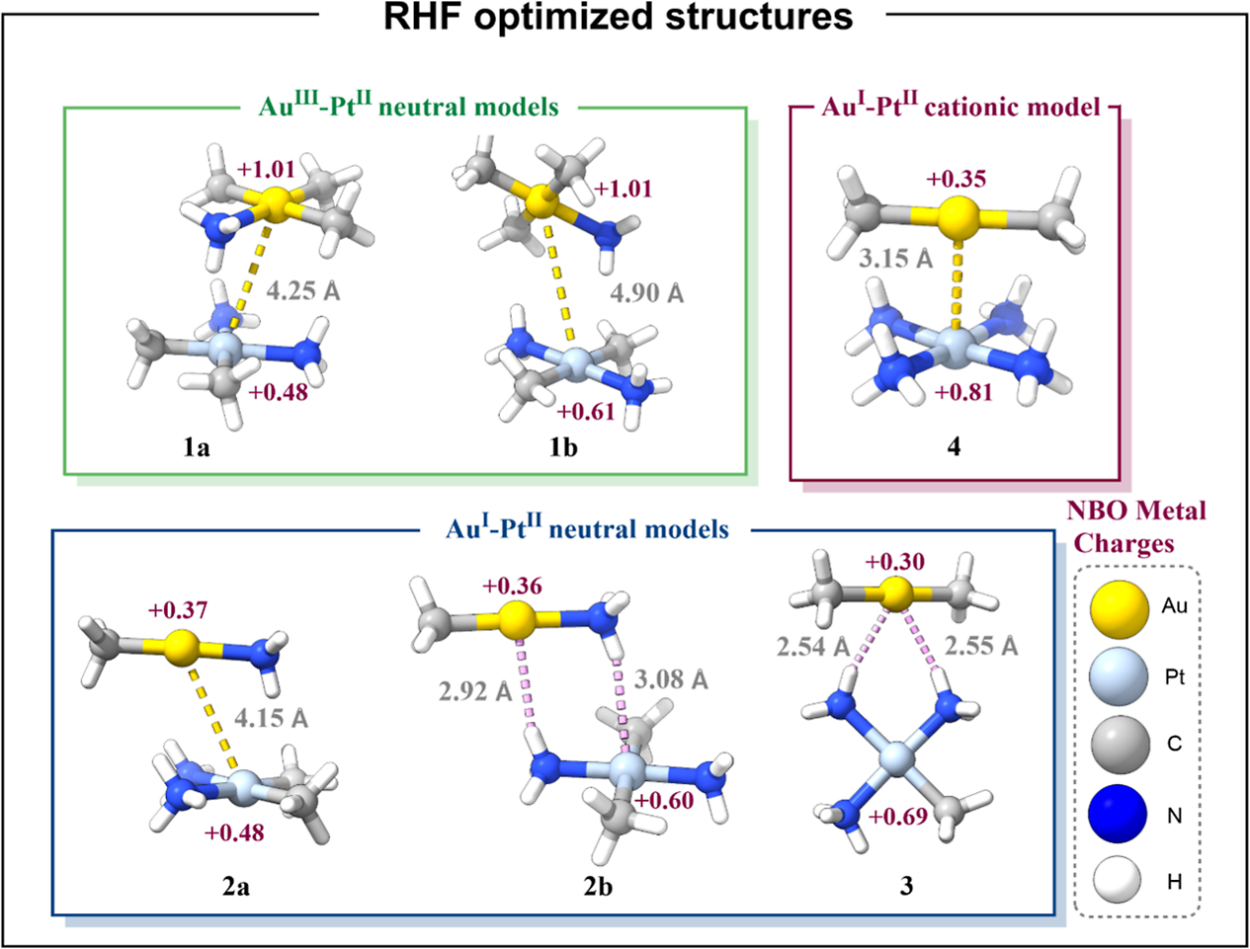
Representations of the RHF/def2-TZVP optimized structures of models **1–4**. The most relevant distances and the NBO metal charges of the optimization are included.

All Au–Pt distances increased compared with the MP2-optimized structures. For models **1a**, **1b**, and **2a**, the structures remain similar, but the metal–metal distances increase by approximately 1 Å, ruling out a metallophilic interaction. For models **2b** and **3**, metal–hydrogen bonds prevail over the metallophilic interactions. This was already the case for model **2b** at the MP2 level of theory; however, for model **3**, the structure changed completely, forming two Au···H–N bonds in the same plane of the molecules. Finally, the Au–Pt distance in model **4** increased by only 0.27 Å due to the strong Coulombic component. It is possible that the metallophilic interaction still exists in this model because the distance is shorter than the sum of van der Waals radii. In light of the comparison between optimizations, the MP2-optimized structures were analyzed.

The neutral models displaying Au^III^···Pt^II^ interactions **1a** (3.51 Å) and **1b** (3.37 Å) exhibited the longest distances, which is consistent with the repulsion caused by the higher positive charges compared with the other models. The neutral models displaying Au^I^···Pt^II^ interactions showed shorter distances than the latter ones. This could be due to the higher electron density of Au^I^ compared to that of Au^III^ and its linear geometry, which results in fewer steric repulsions between ligands. It is noteworthy that model **2b** tends to form hydrogen bonds (Au^I^···H–N and Pt^II^···H–N) instead of a metallophilic interaction, as the intermetallic distance is too long (4.22 Å). For this reason, the same model was optimized with the metal center coordinates frozen (model **2c**) to study the metallophilic interaction and compare it with the previous model. For cationic model **4**, the intermetallic distance of 2.88 Å was surprisingly short, suggesting the strongest interaction among the models, attributable to the combination of Coulombic attraction and a dispersive component. In addition, the optimized geometries facilitate hydrogen bonding contacts between the metals and hydrogen atoms of the ligands, thereby supporting the metallophilic interactions. These types of interactions have been widely investigated both experimentally and computationally.^88,89^

Therefore, the MP2 optimized structures were suitable for potential attractive gold···platinum interactions, and we performed a full computational and topological analysis.

### 3.2 Computational Analysis of the Metallophilic Interactions

The PECs of the optimized structures were computed to obtain the intermetallic distance with the maximum interaction energy. The PECs were obtained by stretching the Au–Pt distance to the selected values, without reoptimization of the rest of the model. The interaction energy was computed at the RHF and MP2 levels of theory with the def2-TZVP basis sets for all atoms. The counterpoise (cp) correction for the BSSE was applied at each point, and they have been fitted using the Herschbach–Laurie four-parameter function. In addition, to study the impact of relativistic effects, which have been demonstrated to influence metallophilic interactions,^5^ calculations were performed using quasi-relativistic (QR), nonrelativistic (NR), and FR ECP for the metal centers. For more details of the PECs calculations, see the Supporting Information. Thus, the cp-corrected RHF and MP2 interaction energies (Δ*E*_int_) as functions of the Au–Pt distances are plotted in Figure 3.

**Figure 3:**
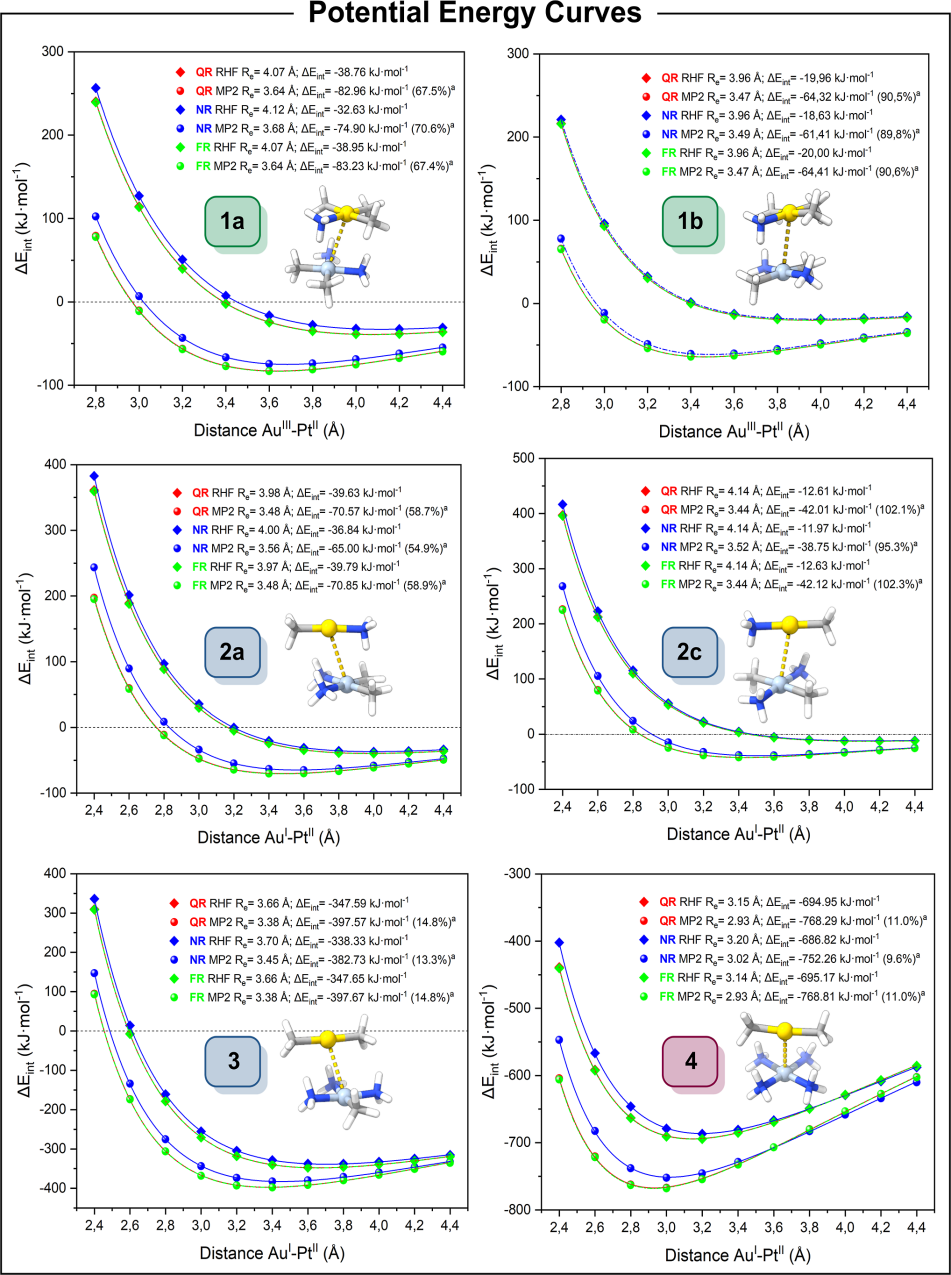
Total interaction energy as a function of the Au–Pt distance calculated at the RHF and MP2 levels of theory with different relativistic ECPs (i.e., QR quasi-relativistic, NR non-relativistic, FR fully relativistic). Raw data are provided in the Supporting Information, Tables S2–S7. ^a^ The electronic correlation contribution was calculated with eq S3.

Different conclusions can be drawn by observing the shapes of the PECs. The attractive or repulsive nature between monomers can be evaluated by examining whether the PECs possess a minimum in the curve, indicating an attractive nature, or not, which would suggest repulsion. The dispersion forces can be estimated by considering how electron correlation is accounted for in the computational framework. Additionally, the influence of relativistic effects can be evaluated by observing the differences among the different ECPs in the PECs.

The calculated PECs of all models exhibited a minimum at both levels of theory, indicating their attractive nature. The interaction energy and the intermetallic distance of this minimum varied depending on the level of theory due to the different treatments of electronic correlation. At the RHF level of theory, electron correlation is not considered, thus it does not accurately describe dispersion interactions (e.g., metallophilic interactions, hydrogen bonds, halogen bonds, among others). In contrast, the MP2 level of theory accounts for this phenomenon. It should be noted that this level of theory tends to overestimate this kind of interactions;^1^ however, it is considered a good indicator of their existence.^30^ The importance of electronic correlation effects is evidenced by the shortening of the distance between the metal centers and energy stabilization at the MP2 level of theory compared to the RHF level of theory. Moreover, the presence of other dispersive interactions within the models should be considered as they contribute to the total interaction energy. These mainly consisted of hydrogen bonds such as Au···H–N, Pt···H–N or C···H–C, C···H–N, or N···H–C interactions.

The PECs of the neutral models **1** displaying Au^III^···Pt^II^ interactions presented minima for both levels of theory. At the RHF level of theory, it is assumed that the ionic interaction is responsible for their attractive behavior. At the MP2 level, model **1a** showed an equilibrium distance of 3.64 Å with an interaction energy of −83 kJ·mol^–1^, while model **1b** revealed an equilibrium distance of 3.47 Å with an interaction energy of −64 kJ·mol^–1^. The difference between MP2 and RHF accounts for the electronic correlation component; see eq S3. Hence, the contribution of the electron correlation effects represents 67% and 91% of the total interaction energy, respectively. These findings are consistent with a mainly dispersive attraction between the metal centers that stabilize the system.^30^ These results are similar to those in our previous study of the d^8^–d^8^ system displaying unsupported Au^III^···Au^III^ interactions, see model **S1**, Supporting Information.^51^

The PECs of neutral models **2–3** displaying Au^I^···Pt^II^ interactions also displayed minima for both levels of theory. There is a significant difference in the magnitude of the interaction energy between the models. Models **2a** and **2c**, consisting of neutral fragments, are similar to Au^III^···Pt^II^ models **1a** and **1b**. At the MP2 level of theory, model **2a** showed an equilibrium distance of 3.48 Å with an interaction energy of −71 kJ·mol^–1^, while model **2c** revealed an equilibrium distance of 3.44 Å with an interaction energy of −42 kJ·mol^–1^. The linear geometry of the gold fragment decreases the steric hindrance and minimizes additional interactions with ligands compared with the planar square geometry of Au^III^ fragments. Thus, these models enable us to study metallophilic interactions in a more focused way. On the other hand, model **3**, which is composed of ionic fragments, showed an equilibrium distance of 3.38 Å and an interaction energy of −398 kJ·mol^–1^. The greater stabilization compared to the other models arises from the ionic attraction due to the opposite charges of the fragments. This is consistent with the lower electronic correlation component percentage (ca. 14%). The PECs for model **2b**, which displays metal–hydrogen bonds, are plotted in Figure 4. It showed a Pt–H distance of 2.40 Å and an interaction energy of −58 kJ·mol^–1^. Thus, each metal–hydrogen bond contributes approximately with 29 kJ·mol^–1^.

**Figure 4:**
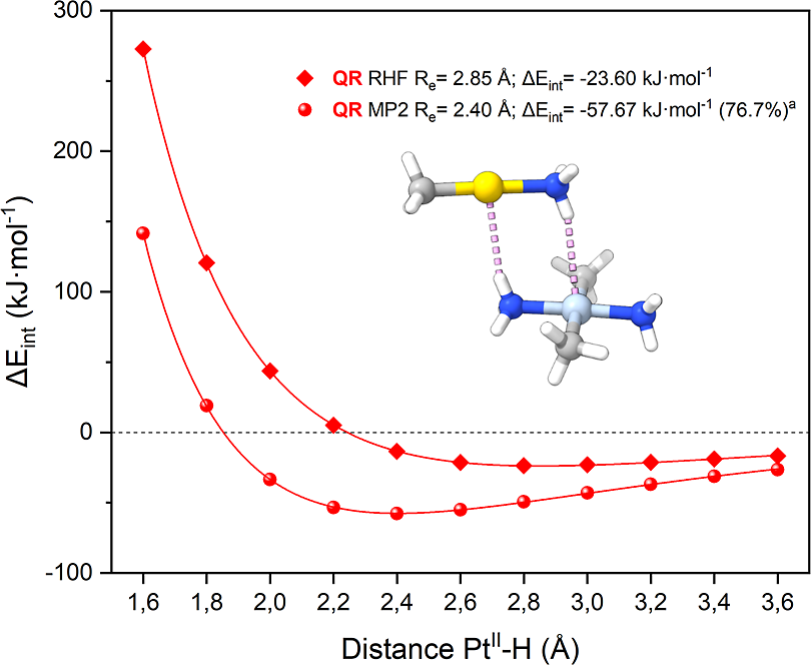
Total interaction energy of model **2b** as a function of the Pt–H distance calculated at the RHF and MP2 levels of theory. Raw data are provided in Table S8.

The PECs for cationic model **4** presented the higher interaction energy in minima for both levels of theory with an equilibrium distance of 2.93 Å and an interaction energy of −768 kJ·mol^–1^. The difference in the magnitude of energy is due to opposite charges of the gold and platinum fragments, which is consistent with the great decrease in the percentage of the electronic correlation component compared to the other models, similar to model **3**.

To refine the interaction energies, we performed counterpoise-corrected calculations at the minima of the PECs using QR ECP at more computationally demanding levels of theory. This approach allows us to compare the suitability of the MP2 level of theory for studying our models with other methods, such as DLPNO-CCSD­(T) or SCS-MP2, which better accounts for electronic correlation. Table 1 presents the results of these calculations and highlights the differences in values compared to those of the MP2 level of theory.

**Table 1:** Counterpoise-Corrected Interaction Energies (kJ·mol^–1^) Calculated at SCS-MP2 and DLPNO-CCSD­(T) Levels of Theory with the def2-TZVP Basis Sets, at the QR-ECP MP2 Minima of the PECs (Å)

model	method	*R*_Au–Pt_	Δ*E*_int_	Δ*E*_MP2_ ^a^
**1a**	SCS-MP2	3.64	–81.55	+1.31
	DLPNO-CCSD(T)		–79.19	+3.67
**1b**	SCS-MP2	3.47	–70.69	–6.37
	DLPNO-CCSD(T)		–68.43	–4.11
**2a**	SCS-MP2	3.48	–74.22	–3.65
	DLPNO-CCSD(T)		–69.93	+0.64
**2b**	SCS-MP2	4.32	–57.93	–0.26
	DLPNO-CCSD(T)		–53.99	+3.68
**2c**	SCS-MP2	3.44	–43.57	–1.56
	DLPNO-CCSD(T)		–37.83	+4.18
**3**	SCS-MP2	3.38	–394.83	+2.74
	DLPNO-CCSD(T)		–391.92	+5.65
**4**	SCS-MP2	2.93	–765.18	+3.11
	DLPNO-CCSD(T)		–757.56	+10.73

a Δ*E*_MP2_ represents the energy difference with respect to the MP2/def2-TZVP level of theory (in kJ·mol^–1^).

The results were somewhat unexpected. One might assume that at both levels of theory, each offering a more accurate and efficient treatment of electron correlation, the interaction energies would be lower compared to those obtained with the MP2 level of theory, given that MP2 is known to overestimate metallophilic interactions. However, this was not the case for some models, where the interaction energy calculated using SCS-MP2, and even CCSD­(T) for model **1b**, was higher than that obtained with MP2 (Δ*E*_MP2_ < 0). It is important to note that the observed overestimation at other theoretical levels, compared to MP2, may be attributed to the fact that we are calculating the minimum of the MP2 curve, which corresponds to the point of maximum interaction for our systems at the MP2 level of theory. Nevertheless, the values of Δ*E*_MP2_ are relatively low, indicating that the MP2 level of theory is well-suited for studying metallophilic interactions in our computational models and is consistent with results from more accurate computational levels.

Finally, relativistic effects were evaluated by using different ECPs. The differences between the interaction energy considering relativistic effects (FR) and without them (NR) were calculated employing eq S4. This difference ranged from 2 to 10%. Hence, relativistic effects exhibit lower intensity in the Au···Pt interaction compared to the Au^I^···Au^I^ one or Au^I^···Hg^II^, where they contributed with a 22–27% to the total interaction energy.^29,47^ This is expected, since Au^III^ and Pt^II^ cations have less significant relativistic effects than the Au^I^ cation.^90^ Additionally, they exhibit relativistic effects of similar intensity compared to model **S1**, which displays Au^III^···Au^III^ interactions (see Figure S1).·The equilibrium distance is slightly longer for NR curves compared to the QR-ECP and FR curves, which is in agreement with the influence of this phenomenon on metallophilic interactions. The fact that the nonrelativistic RHF minima occurs at a considerable distance (ca. 4 Å) strongly implies that relativistic effects play a significant role in both the Coulombic and electronic correlation components of the interactions. Moreover, the difference in using a QR-ECP or an FR one in terms of the interaction energy and intermetallic distance is practically negligible.

### 3.3 NBO, Bond Indices, and NEDA Analysis

We computed the NBO and the QTAIM effective charges at the minima of the PECs at the MP2/def2-TZVP level of theory with QR-ECP. The metal charges for both methods are listed in Table 2, with the most significant ones detailed in Tables S10 and S11.

**Table 2:** Effective Charges of the Metal Centers, Bond and Penetration Indices for the Metal–Metal Interactions Calculated at the MP2/def2-TZVP Level of Theory^a^

model	Au *q* ^NBO^	Pt *q* ^NBO^	Au *q* ^QTAIM^	Pt *q* ^QTAIM^	WBI	IBSI	DI	*p*_AuPt_
**1a**	+0.79 (+0.82)	+0.20 (+0.21)	+0.36 (+0.48)	+0.21 (+0.19)	0.07	0.01	0.08	51.4
**1b**	+0.79 (+0.82)	+0.30 (+0.33)	+0.43 (+0.48)	+0.36 (+0.36)	0.10	0.02	0.13	59.4
**2a**	+0.26 (+0.23)	+0.17 (+0.21)	+0.04 (+0.02)	+0.22 (+0.19)	0.11	0.02	0.13	59.7
**2c**	+0.25 (+0.23)	+0.27 (+0.33)	+0.03 (+0.02)	+0.36 (+0.36)	0.12	0.02	0.16	61.9
**3**	+0.24 (+0.20)	+0.34 (+0.38)	–0.06 (−0.07)	+0.44 (+0.44)	0.12	0.02	0.15	65.0
**4**	+0.27 (+0.20)	+0.41 (+0.59)	+0.01 (−0.07)	+0.67 (+0.74)	0.27	0.06	0.35	88.8

a The charges of the atoms in their respective monomers are shown in parentheses.

The NBO charge distribution in models **1a** and **1b** indicates that the gold center has a charge of +0.79, which is significantly lower than that in its formal oxidation state of +3. In the other models, the charge on the gold center is approximately +0.25, indicating a significant reduction from its formal oxidation state of +1. Within the QTAIM framework, the effective metal charges decrease significantly: for models **1a** and **1b**, the charges are reduced to +0.36 and +0.43, respectively, while for the remaining models, the effective metal charges range from +0.04 to −0.06. For the platinum center, the charges range from +0.17 to +0.67 for both methods, showing a notable decrease compared with its formal oxidation state of +2. These findings emphasize that electron-donor ligands play a crucial role in reducing electrostatic repulsion between cationic centers, thereby promoting metallophilic interactions. A comparison of coordinated and free molecules shows significant metal-to-metal and ligand-to-metal charge transfer, particularly in the platinum fragments, with model **4** exhibiting the strongest effect. In conclusion, the presence of electron-donating ligands enhances the formation of metallophilic interactions.

The bond order measures the number of pairs of electrons shared between atoms and directly reflects the degree of delocalization of the electrons between two atomic spaces. Hence, we calculated the Wiberg bond index (WBI) and the intrinsic bond strength index (IBSI) between the metals, to gain further insight into the strength of the metallophilic interactions, see Table 2. The low bond index values indicate noncovalent interaction between the metal centers. Both bond orders follow the same trend and are consistent with the previous analyses discussed. Model **1a**, with the largest gold–platinum distance, shows the lowest bond order, while models **1b** and **2–3** display comparable values. The highest bond order value is observed in model **4**, which has the shortest gold–platinum distance. Additionally, we computed the WBI and IBSI of the other BCPs (vide infra) present in the models, see Tables S12–S16. Several metal–hydrogen bonds (Au···H–N or Pt···H–N) are found but with lower bond order values compared to their corresponding metal–metal interactions. Hence, the metallophilic interactions are the strongest among the noncovalent interactions in these models.

Additionally, we computed the delocalization index (DI) within the QTAIM framework, which quantifies the number of electron pairs shared or delocalized between two atomic basins. In closed-shell systems, such as those studied here, the DI serves as a reliable indicator of electron delocalization between interacting atoms. The obtained values are consistent with those of the computed bond orders. The low DI values between the metal centers reflect a limited degree of electron sharing, supporting the interpretation of these contacts as primarily dispersive in nature. Notably, model **4** displays a significantly higher DI compared to the others, in agreement with the previously discussed trends.

The recently proposed penetration index (*p*_AB_),^91^ which measures the ratio between the intersection of the two van der Waals crusts and the sum of their crust widths (see eq S5), was calculated to characterize the gold–platinum interactions. These values provide a qualitative indication of the strength of the bonds or interactions. Briefly, values of *p*_AB_ larger than 100% indicate ionic or covalent bonding; conversely, values of 0% denote that the distances between the atom pairs are equal to the sum of their van der Waals radii. Therefore, we expect that values around 0% correspond to van der Waals interactions, and values above 100% correspond to covalent bonds. The distribution of aurophilic interaction penetration indices ranges from 40% to 80%, with a maximum at 68%. For our models, the *p*_AuPt_ varied from 51.4% to 65.0%, consistent with the typical values for metallophilic interactions. The penetration increased across the models, following the same trend observed in PECs and bond order analyses. Notably, model **4** stood out with the highest *p*_AuPt_ (88.8%), attributed to the contribution of Coulombic attraction. Similar cases of Pt···Pt and Pd···Pd interactions with penetrations exceeding 90% have been reported, driven by strong electrostatic attraction between ions.^92,93^

We therefore carried out a NEDA at the PBE0-D3­(BJ)/def2-TZVP level of theory to elucidate the physical origin of the attractive interactions. This analysis provides the partition of the molecular interaction energies, including charge transfer, electrical interaction (polarization effects, Coulombic and dipole–dipole interactions), and core repulsion (electron exchange and correlation effects, self-polarization energy, and Pauli repulsion). We focus on trends across our models rather than providing absolute values, as, to the best of our knowledge, this analysis cannot be performed at the MP2 level of theory in the Gaussian 16 program. Thus, the most important components of the analysis are plotted at Figure 5, all values are listed at Table S17, and the NEDA theory is included in the Supporting Information.

**Figure 5:**
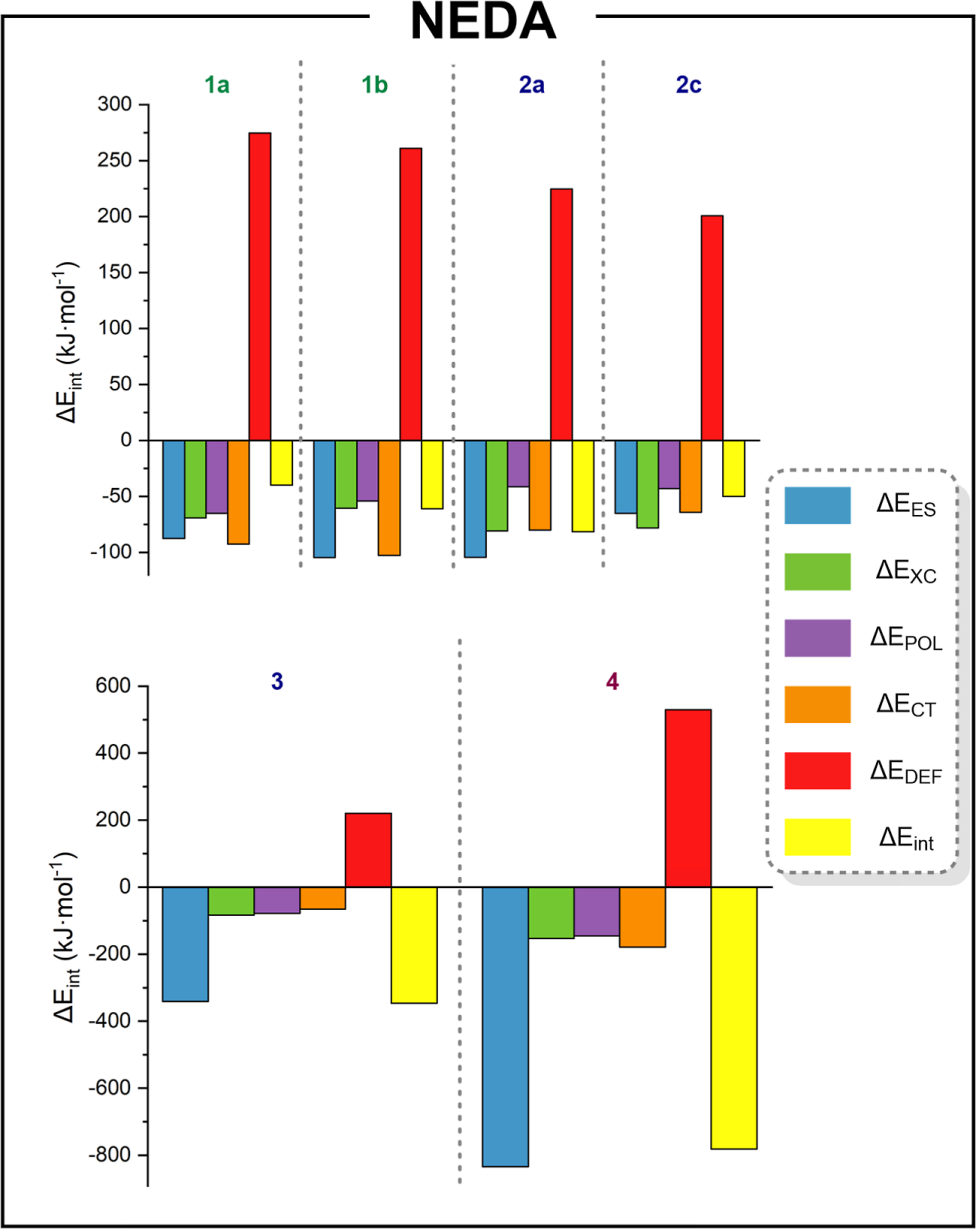
NEDA plots at the PBE0-D3­(BJ)/def2-TZVP level of theory. Energy values are given in kJ·mol^–1^. Raw data are provided in Table S17.

The interaction energies (Δ*E*_int_) follow the tendency of a previous analysis (i.e. **2c** < **1b** < **2a** < **1a** ≪ **3** ≪ **4**). It is important to note that these values are not quantitatively comparable to the MP2 PECs analysis, as NEDA uses the DFT hybrid functional PBE0 with dispersion correction D3­(BJ). The perturbative MP2 method naturally captures dispersion interactions through electron correlation, with a decay of *R* ^–6^, reflecting their physical origin. In contrast, DFT requires empirical dispersion corrections using fitted parameters to approximate long-range interactions. Standard DFT functionals often fail to accurately describe dispersion, exhibiting an unphysical *R* ^–3^ decay. Grimme’s corrections attempt to address this by adding terms but rely on damping functions. This explains the differences in the energy values.

The partitioning of molecular interactions provides interesting results. In the neutral models **1a** and **1b**, which display Au^III^···Pt^II^ interactions, the electrostatic (Δ*E*_ES_), charge transfer (Δ*E*_CT_), and electron exchange and correlation effects (Δ*E*_XC_) are the predominant components, each accounting for approximately 25% to 30% of the total interaction energy. Notably, the Pauli repulsion (Δ*E*_DEF_) component is the largest one, a common characteristic in other metallophilic systems.^34^

In neutral models **2a** and **2c**, which display Au^I^···Pt^II^ interactions, the distribution is similar to that of the latter ones. However, the Pauli repulsion component decreases due to reduced hindrance between ligands, resulting from the lower coordination index in the gold fragment. Conversely, in model **2b**, which contains metal–hydrogen bonds and no metallophilic interaction, the charge transfer component is the main contribution with the 44% of the total interaction energy, and the polarization effects decreases to only 3%, see Table S17.

The electrostatic component represents approximately 60% of the total interaction energy in models **3** and **4**, whereas the charge transfer component decreases to 11.5% and 13.6%, respectively. Thus, the main contribution in these models is the Coulombic attraction between the anions, which is consistent with the PECs analysis.

### 3.4 Topological Analysis of the Electron Density

A combined QTAIM/IGMH analysis of the electron density was carried out to better understand the Au···Pt interactions. All calculations were performed on the geometries of the PECs minima at the MP2/def2-TZVP level of theory with QR-ECP.

According to the QTAIM approach, there is a bond critical point (BCP) of the electron density between any pair of bonded atoms in a molecule.^76^ Additionally, there is a bond path with the maximal gradient path linking the nuclei in question. It should be noted that the physical meaning of bond paths is controversial; it represents more than just Lewis-type bonds, encompassing a broader concept that also includes dispersion and van der Waals interactions.^94,95^ The values of the local properties at the BCPs are related to the nature and strength of the bonding interactions between atoms, and this approach is frequently used in the investigation of different type of interactions.^26,28,39,45,96,97^

For all models, (3, −1) characteristic BCPs were found along the bond path between the gold and platinum atoms. The results of the QTAIM properties of these BCPs are listed in Table 3.

**Table 3:** Properties of the QTAIM (3, −1) Bond Critical Points between Gold and Platinum Atoms Calculated with the MP2/def2-TZVP Electron Density^a^

model	*r*_e_	sign(λ_2_)·ρ_e_(*r*)	∇^2^ρ_e_(*r*)	*H*(*r*)·10^3^	*G*(*r*)	*V*(*r*)	|*V*(*r*)|/*G*(*r*)	*G*(*r*)/ρ_e_	*E*_int_ ^b^	*E*_int_ ^c^
**1a**	3.638	–0.0121	0.0286	–0.1178	0.0073	–0.0074	1.0162	0.5984	9.69	8.19
**1b**	3.470	–0.0153	0.0361	–0.5190	0.0095	–0.0101	1.0544	0.6235	13.20	10.74
**2a**	3.481	–0.0158	0.0349	–0.7632	0.0095	–0.0102	1.0805	0.6002	13.45	10.68
**2c**	3.441	–0.0161	0.0371	–0.7046	0.0100	–0.0107	1.0706	0.6206	14.03	11.24
**3**	3.381	–0.0181	0.0419	–1.0453	0.0115	–0.0126	1.0907	0.6364	16.50	12.98
**4**	2.933	–0.0391	0.0981	–5.6397	0.0301	–0.0357	1.1873	0.7693	46.92	33.91

a M–M distance (*r*_e_ in Å), product of the sign of second largest eigenvalue of Hessian matrix of electron density (sign­(λ_2_)·ρ_e_(*r*) in au), Laplacian of electron density (∇^2^ρ_e_(*r*) in au), electron energy density (*H*(*r*) in au), Lagrangian kinetic energy density (*G*(*r*) in au), potential energy density (*V*(*r*) in au), ratio |*V*(*r*)|/*G*(*r*), ratio |*V*(*r*)|/2*G*(*r*), ratio *G*(*r*)/ρ_e_ in au, estimated energies (*E*_int_ in kJ·mol^–1^).

b *E*_int_ = −*V*(*r*)/2.

c *E*_int_ = 0.429*G*(*r*).

By considering the QTAIM properties, one can investigate the nature and strength of the BCPs. Thus, large electron density ρ_e_(*r*) values (i.e. >0.2 au), along with a negative Laplacian of the electron density ∇^2^ρ_e_(*r*) and negative electron energy density *H*(*r*), indicate accumulated electron density, characteristic of shared shell interactions (e.g., covalent bonds). Conversely, small ρ_e_(*r*) values (i.e. <0.1 au), a positive ∇^2^ρ_e_(*r*) and positive *H*(*r*), suggest a reduced electron density, typical of closed shell interactions (e.g., ionic, hydrogen bonding, van der Waals).^98^ The ∇^2^ρ_e_(*r*) and *H*(*r*) values listed in Table 3 have opposite signs, indicating an intermediate between an ionic and covalent interaction. This suggests that the Au···Pt interactions are not purely ionic but also exhibit electron-sharing characteristics. Furthermore, the low ρ_e_(*r*) values support a closed shell type interaction with donor–acceptor properties.^99^ According with the ρ_e_(*r*) at the BCPs, the interaction strength follows **1a** < **1b** < **2a** < **2c** < **3** < **4**, which is in good agreement with the observations of the previous analyses.

A sharper classification can be achieved by using the Lagrangian kinetic energy density *G*(*r*) and the potential energy density *V*(*r*) properties. When *V*(*r*) locally dominates, it indicates charge concentration, characteristic of shared shell interaction. However, when *G*(*r*) dominates, it indicates charge depletion, which is typical of closed shell interactions. With the ratio |*V*(*r*)|/*G*(*r*), one can distinguish three categories of closed shell interactions based on the degree of covalency. If this ratio is smaller than 1, the leading term is *G*(*r*) and electrons are destabilized close to the BCP indicating no covalency, which is characteristic of pure closed shell interactions (pure CS). When this ratio is higher than 2, *V*(*r*) is large and the electrons are stabilized at the BCP, suggesting electron sharing (covalency). If 1 < |*V*(*r*)|/*G*(*r*) < 2, the interaction exhibits an intermediate character, as it is not purely ionic and denotes some degree of covalent character. Nakanishi and Hayashi referred to this as regular closed shell (regular CS) interactions.^100^ The ratio |*V*(*r*)|/*G*(*r*) values listed in Table 3 are close to 1, suggesting a regular CS type interaction, which is in good agreement with the previous QTAIM properties. Additionally, the degree of covalency increases from model **1** to **4**.

Based on ratio *G*(*r*)/ρ_e_, Macchi et al. categorized closed shell interactions as metallic (shared) bonding and donor–acceptor interactions.^101^ Thus, heavy metal interactions that form metallic bonds are characterized by *G*(*r*)/ρ_e_ < 1, whereas closed shell donor–acceptor interactions are defined by *G*(*r*)/ρ_e_ ≈ 1. Thus, the models show metallic bond values that increase progressively toward closed shell donor–acceptor interactions from **1** to **4**, following a similar trend as covalency.

Finally, we estimated the metallophilic interactions energy using Espinosa and Vener approaches.^102,103^ The interaction energy ranges from 9.7 to 46.9 kJ·mol^–1^ in the Espinosa method and from 8.2 to 33.9 kJ·mol^–1^ in the Vener approach. Models **1** to **3** exhibit values characteristic of metallophilic interactions (ca. 15 kJ·mol^–1^); however, model **4** displays higher values due to the strong Coulombic attraction. Moreover, the interaction energy values follow the same trend as that of the ρ_e_(*r*) property.

Briefly, the QTAIM analysis (i) reveals a BCP with the corresponding bond path for all models, (ii) the QTAIM properties suggest a regular closed shell interaction with some electron sharing as metallic bond, (iii) the interaction energy magnitudes indicate weak interactions (like van der Waals forces), (iv) analysis of the ρ_e_ at BCP reveals that the Au···Pt interaction exhibits a lower ρ_e_ value than typical Au^I^···Au^I^ interactions (ρ_e_ = 0.0217–0.0274 e·Å^–3^),^97^ yet significantly higher than those reported for Au^III^···Au^III^ ones (ρ_e_ = 0.0036–0.0078 e·Å^–3^).^19,51^ These values suggest that the Au···Pt interaction lies intermediate in strength between these two cases. Moreover, the positive sign of the Laplacian of the electron density (∇^2^ρ > 0) at the BCP is consistent across all three types of interactions, corroborating their classification as closed-shell, noncovalent in nature.

The independent gradient model based of Hirshfeld partition of the molecular density (IGMH) method is a recently proposed real-space function based on the reduced density gradient (RDG), which is a powerful tool to visualize and analyze covalent and noncovalent interactions.^84^ This method offers key advantages compared to the very popular NCI method,^104^ such as the availability of multiple quantitative indices, the definition of fragments to separate intra- and intermolecular interactions, and clearer interaction isosurfaces.

Thus, the RDG can show positive and high values, characteristics of regions far from the molecule, and values close to zero where both covalent and noncovalent interactions appear. Regions with low gradient and low density indicate the presence of weak interactions, while regions with high density and low gradient indicate stronger interactions. In addition, the sign of the second largest eigenvalue (λ_2_) of Hessian of the electron density (ρ_e_) is considered. Positive λ_2_ values correspond to repulsive interactions, while negative λ_2_ values correspond to attractive interactions. These parameters are represented as an isosurface weighted by the sign­(λ_2_)·ρ_e_(*r*), displaying noncovalent interactions as broad regions. The isosurfaces are colored according to the Blue–Green–Red (BGR) scheme. One can distinguish attractive (blue to green, sign­(λ_2_)·ρ_e_(*r*) < 0), van der Waals (sign­(λ_2_)·ρ_e_(*r*) ≈ 0), and repulsive (green to red, sign­(λ_2_)·ρ_e_(*r*) > 0) interactions.

The mapped IGMH isosurfaces combined with the BCPs, ring critical points, and bond paths of the QTAIM analysis are shown in Figure 6.

**Figure 6:**
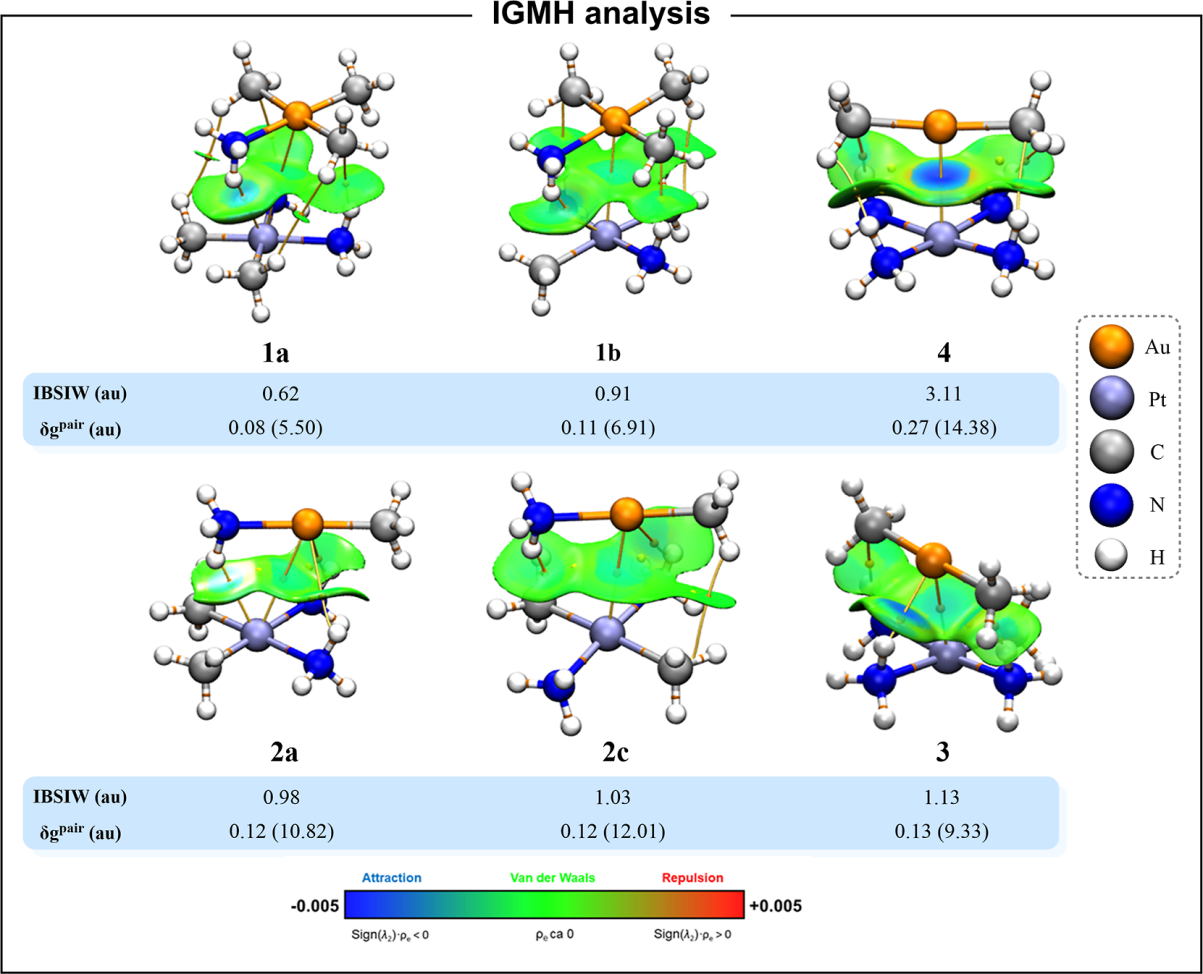
QTAIM (3, −1) BCPs (orange dots), (3, +1) RCPs (yellow dots), bond paths (yellow strings) and the IGMH isosurface (isovalue = 0.005 au).

A bluish spot between the metals was found in all models, corresponding to an electron-rich area (ρ_e_(*r*) > 0) with attractive bonding behavior (λ_2_ < 0). These observations, along with the BCPs and bond paths, are the ultimate topological proof of the existence of an attractive interaction between Au and Pt metals. Additional bluish spots and bright green areas were found involving M···H–N and M···H–C interactions. The rest of green areas are the expected region for van der Waals interactions between ligands. Additionally, we computed the IRI isosurface, which displays intra- and intermolecular interactions; see Figure S2. The results are similar to the IGMH methodology.

We considered some IGMH indices to quantify the Au···Pt interaction. The atomic pair δ*g* index (δ*G* ^pair^) quantifies the contribution of an atom pair to the interaction between two specific fragments. The atomic δ*g* index (δ*G* ^atom^) serves as a quantitative measure of the importance of an atom to interfragment interaction. The intrinsic bond strength index for weak interactions (IBSIW), inspired by the IBSI, showed the ability to distinguish interaction strengths. Thus, the larger the index, the stronger interaction. The δ*G* ^pair^ and IBSIW values are depicted in Figure 6, while δ*G* ^atom^ values are listed in Table S18. δ*G* ^pair^ and IBSIW values confirm the strength order of **1a** < **1b** < **2a** < **2c** < **3** < **4**, as previous analyses. δ*G* ^atom^ shows that the gold atom is more involved in the interaction than platinum, except for model **1a**, which displays the weakest interaction.

By studying these quantitative indices for the additional interactions (displaying BCPs in QTAIM analysis), we observed that M···H–N interactions are similar in strength to, or even stronger than, M···M interactions (Tables S19–S24). Thus, this kind of interaction reinforces the latter, especially for Au^III^ models due to its square planar geometry. The analysis of model **2b** reveals the competition between the hydrogen bond and metallophilic interactions in stabilizing the system, see Figure S3.

## 4 Conclusions

In summary, we have demonstrated the dispersive nature of the uncommon, unsupported Au^III^···Pt^II^ and Au^I^···Pt^II^ interactions in computational models based on experimental complexes. Through computational and topological analysis, these interactions are categorized as a regular closed shell with some degree of electron-sharing. Their strength, around 15 kJ·mol^–1^, is notably lower than that typically reported for the well-known Au^I^···Au^I^ interactions (20–50 kJ·mol^–1^).

This difference in interaction strength can be attributed to several factors, including the mismatch in metal type and oxidation state, which limits the complementarity and enhances the asymmetry of the interaction. The use of electron-donating ligands, such as nitrogen- or carbon-based donors, is essential to mitigate electrostatic repulsion arising from the high effective charge of metal centers, particularly in the cases of Au^III^ and Pt^II^. Furthermore, the influence of relativistic effects in Au···Pt contacts is found to be moderate (2–10%), whereas in Au^I^···Au^I^ systems such effects are known to contribute more significantly to interaction stabilization (22–27%). It is important to note that other types of interactions, such as hydrogen bonding, π-stacking, or ionic attraction, not only reinforce the stability of the systems but may also compete with them.

This study highlights the strong qualitative agreement between different numerical and topological electron density methods in characterizing metallophilic interactions. Moreover, the computational findings provide guidelines for future synthetic work with potential applications that are now under progress.

## Supplementary Material



## Notes

Computational details, optimized structures, interaction energy calculations, NBO effective charges, bond orders, NEDA, IGMH, IRI analysis, and Cartesian coordinates of models **1–4** (PDF)

## †

CEITEC—Central European Institute of Technology, Masaryk University, Kamenice 5/A4, 62500 Brno, Czechia

## Abbrevations

BCPs: bond critical points
BSSE: basis set superposition error
cp: counterpoise correction
D3BJ: third-generation dispersion correction with Becke–Johnson damping
def2-TZVP: triple-zeta valence polarization Karlsruhe basis sets
DFT: density functional theory
DI: delocalization Index
DPLNO-CCSD­(T): domain-based local pair natural orbital coupled cluster with single, double, and perturbative triple excitations
ECPs: effective core potentials
FR-ECP: fully-relativistic effective core potential
IBSI: intrinsic bond strength index
IBSIW: intrinsic bond strength index for weak interactions
IGMH: independent gradient model based on Hirshfeld partition analysis
IRI: interaction region indicator
MP2: second-order Møller–Plesset perturbation theory
NBO: natural bond orbital
NCI: non-covalent Interactions
NEDA: natural energy decomposition analysis
NR-ECP: non-relativistic effective core potential
PBE0: Perdew–Burke–Ernzerhof hybrid functional
PECs: potential energy curves
QTAIM: quantum theory of atoms in molecules
QR-ECP: quasi-relativistic effective core potential
RCP: ring critical point
RDG: reduced density gradient
RHF: restricted Hartree–Fock theory
RI: resolution of identity approximation
SCS-MP2: spin-component-scaled variant of second-order Møller–Plesset perturbation theory
WBIs: Wiberg bond indices
