# Supplementary material for: Unraveling Unsupported Gold···Platinum Metallophilic Interactions: An In-Depth Computational and Topological Study
Source: Inorg Chem. 2025 Jun 25;64(34):17121–34. doi: 10.1021/acs.inorgchem.5c01042 (PMC12406201; doi:10.1021/acs.inorgchem.5c01042)
Supplement: Supplementary file 1 [file ic5c01042_si_001.pdf]

Supporting Information

Unraveling Unsupported Gold···Platinum Metallophilic Interactions: An In-Depth Computational and Topological Study†

Félix Reboiro,[a] Daniel Blasco,[a] M. Elena Olmos,[a] José M. López-de-Luzuriaga*[a] and Miguel Monge*[a]

[a] Departamento de Química, Instituto de Investigación en Química de la Universidad de La Rioja (IQUR), Madre de Dios 53, 26006 Logroño, La Rioja, Spain.

E-mail: josemaria.lopez@unirioja.es, miguel.monge@unirioja.es

Electronic Supplementary Information (ESI)

Table of content

Computational Methods 1

Optimized Structures 4

Potential Energy Curves 6

Effective charges 15

Bond orders 17

NEDA 19

IGMH analysis 20

IRI analysis 23

Cartesian coordinates 24

Computational Methods

All models were fully optimized using the Gaussian 16 Revision C.01 suite of programs[1] at the MP2 and RHF levels of theory[2-4] with the Karlsruhe def2-TZVP basis sets[5] for all atoms, 2f-type polarization functions and a 60-electron effective core potential for gold and platinum atoms (def2-ECP).[6] The frequency analyses at the same level of theory were carried out to verify that the optimized geometries belong to minima (no imaginary frequencies were found). Simplified models **1**–**4** were built from scratch and fully optimized without geometrical constraints, except for model **2c**. This model was optimized by freezing the coordinates of the metal centers with the purpose of preserving the metallophilic interaction. The structures were visualized and rendered using GaussView 6.1[7] and UCSF ChimeraX 1.3 visualization programs.[8]

Interaction energies were obtained at the MP2 and RHF levels of theory using eqn (**S1**) using Gaussian 16 program. Therefore, a counterpoise correction (cp) to the basis set superposition error (BSSE) on interaction energies was performed,[9]

$$\Delta E_{int} = E_{AB}^{(AB)} - E_{A}^{(AB)} - E_{B}^{(AB)} \qquad \textbf{\textit{S1}}$$

where ΔE_{int} represents the interaction energy, $E_{AB}^{(AB)}$ is the energy of the dimer and $E_{A}^{(AB)}$, $E_{B}^{(AB)}$ are monomer´s energies calculated using the basis sets of the dimer. The calculated points were fitted using a four-parameter (eqn(**S2**)), which had been previously used to derive the Herschbach-Laurie relation,[10]

$$\Delta E_{int}\,(R) = Ae^{-BR} - C \cdot R^{D} \qquad \textbf{\textit{S2}}$$

where R is the corresponding interatomic distance and A, B, C, D are fitting parameters. The internal coordinate corresponding to the interatomic distance was manually stretched to the selected values whereas all other internal coordinates were kept intact. Thus, the potential energy curves (PECs) were derived in this manner.

The electronic correlation contribution to the total interaction energy at the minima of the PECs was calculated by subtracting the interaction energy at the MP2 level of theory at the MP2 minimum distance ($\Delta E^{MP2}_{\min MP2}$) from the interaction energy at the RHF level of theory at the MP2 minimum distance ($\Delta E^{RHF}_{\min MP2}$) and dividing it by the first term, as shown in eqn (**S3**).

$$electronic\ correlation\ contribution = \frac{\Delta E^{MP2}_{\min MP2} - \Delta E^{RHF}_{\min MP2}}{\Delta E^{MP2}_{\min MP2}} \cdot 100 \qquad \textbf{\textit{S3}}$$

To evaluate the implications of relativistic effects on interactions, additional PECs were calculated employed non-relativistic (ECP60MHF)[11] and fully relativistic (ECP60MDF)[12,13] 60-electron effective core potentials. Besides, a single-point calculation was performed for all PECs minima at both levels of theory to further refine the interaction energies. Single-point counterpoise-corrected calculations at the SCS-MP2[14] and DLPNO-CCSD(T)[15] levels of theory using the def2-TZVP basis sets were performed in the ORCA 5 software package.[16] The RIJCOSX[17] approximation and the corresponding default auxiliary basis sets for def2-TZVP were employed.

The relativistic effects contribution at each level of theory were calculated according to eqn (**S4**),

$$relativistic\ contribution = \frac{\Delta E_{int}^{FR} - \Delta E_{int}^{NR}}{\Delta E_{int}^{FR}} \cdot 100 \qquad \textbf{S4}$$

where ΔE_{int}^{FR} is the interaction energy at the PEC minima with the fully relativisitic potential and ΔE_{int}^{NR} represents the non-relativistic one.

NBO analysis[18] and Wiberg Bond Index (WBI)[19] calculations were carried out in each computational model using Gaussian 16 at the MP2/def2-TZVP level of theory, with the aim of computing the effective charges within the metal centers and their bonded atoms. Additionally, the Intrinsic Bond Strength Index (IBSI)[20], Delocalization Index (DI)[21] and QTAIM charges[22] were computed at the same level of theory by the Multiwfn 3.8 package.[23]

The penetration index (p_{AB}), recently proposed by Santiago Álvarez *et al.*,[24] was calculated using eqn (**S5**),

$$p_{AB}(\%) = \frac{(v_A + v_B - d_{AB})}{(v_A + v_B - r_A - r_B)} \cdot 100 \qquad \textbf{S5}$$

where v is the corresponding van der Waals radii, r the corresponding covalent radii and d the interatomic distance in the computational models. Additionally, the covalent and van der Waals radii proposed by the same authors were employed.[25,26]

Natural energy decomposition analysis (NEDA)[27,28] was carried out for all models using Gaussian 16 and NBO 7.0 programs.[29] Calculations were performed at the DFT[30,31] level of theory with the Perdew–Burke-Ernzerhof (PBE0) hybrid functional[32] (gaussian keyword PBE1PBE1) using the third empirical dispersion correction by Grimme D3(BJ).[33,34] It allows for the splitting of the interaction energy between monomers into electrical interaction (E_{EI}), charge transfer (E_{CT}) and core repulsion (E_{CORE}) components (eqn (**S6**)).

$$E_{int} = E_{CT} + E_{CORE} + E_{EL} \qquad \textbf{S6}$$

The core repulsion component (E_{CORE}) encompasses the sum of the Pauli repulsion (E_{DEF}), electron exchange and correlation effects (E_{EX}) minus the self-polarization energy (E_{ES}) of the components (eqn (**S7**)).

$$E_{CORE} = E_{DEF} + E_{XC} - E_{SE} \qquad \textbf{S7}$$

The electrical interaction (E_{EL}) is represented by the sum of the electrostatic components (E_{ES}), the sum of the polarization effects (E_{POL}) and the self-polarization (E_{ES}) energy of the components, which the latter is destabilizing in nature. The percentages of the stabilizing components were calculated by dividing the corresponding attractive component by the sum of all the others (eqn (**S8**)).

$$\% = \frac{|E_{CT}| \cdot |E_{ES}| \cdot |E_{POL}| \cdot |E_{XC}|}{E_{CT} + E_{ES} + E_{POL} + E_{XC}} \cdot 100 \qquad \textbf{S8}$$

A topological analysis on each computational model was performed for the porpoise of analyzing the interaction nature from a qualitative point of view. The topology and properties of the MP2/def2-TZVP electron density of the structures have been examined using Quantum Theory of Atoms In Molecules (QTAIM),[22] Interaction Region Indicator (IRI)[35] and Independent Gradient Model based on Hirsfeld partition (IGMH)[36] methods using Multiwfn 3.8 software. VMD 1.9.4a53 visualization program package[37] was employed for the representations of the electron density studied in each analysis.

References

1. M. J. Frisch, G. W. Trucks, H. B. Schlegel, G. E. Scuseria, M. A. Robb, J. R. Cheeseman, G. Scalmani, V. Barone, G. A. Petersson, H. Nakatsuji, X. Li, M. Caricato, A. V. Marenich, J. Bloino, B. G. Janesko, R. Gomperts, B. Mennucci, H. P. Hratchian, J. V. Ortiz, A. F. Izmaylov, J. L. Sonnenberg, D. Williams-Young, F. Ding, F. Lipparini, F. Egidi, J. Goings, B. Peng, A. Petrone, T. Henderson, D. Ranasinghe, V. G. Zakrzewski, J. Gao, N. Rega, G. Zheng, W. Liang, M. Hada, M. Ehara, K. Toyota, R. Fukuda, J. Hasegawa, M. Ishida, T. Nakajima, Y. Honda, O. Kitao, H. Nakai, T. Vreven, K. Throssell, J. A. Montgomery, Jr., J. E. Peralta, F. Ogliaro, M. J. Bearpark, J. J. Heyd, E. N. Brothers, K. N. Kudin, V. N. Staroverov, T. A. Keith, R. Kobayashi, J. Normand, K. Raghavachari, A. P. Rendell, J. C. Burant, S. S. Iyengar, J. Tomasi, M. Cossi, J. M. Millam, M. Klene, C. Adamo, R. Cammi, J. W. Ochterski, R. L. Martin, K. Morokuma, O. Farkas, J. B. Foresman and D. J. Fox, *Gaussian 16 Rev. C.01*, Wallingford CT, 2016.
2. C. Møller and M. S. Plesset, *Phys. Rev.,* 1934, **46**, 618.
3. M. J.Frisch and M. Head-Gordon.; J. A. Pople, *Chem. Phys. Lett.*, 1990, 166, 275.
4. C. C. J. Roothaan, *Rev. Mod. Phys.*, 1951, **23**, 69.
5. F. Weigend and R. Ahlrichs, *Phys. Chem. Chem. Phys.,* 2005, **7**, 3297.
6. D. Andrae, U. Häußermann, M. Dolg, H. Stoll and H. Preuß, *Theor. Chim. Acta* 1990, **77**, 123.
7. R. Dennington, T. A. Keith and J. M. Millam, *GaussView Version 6*, Semichem Inc., Shawnee Mission, KS, **2016**.
8. E. F. Pettersen, T. D. Goddard, C. C. Huang, E. C. Meng, G. S. Couch, T. I. Croll, J. H. Morris and T. E. Ferrin, *Protein Sci.,* 2020, **30**, 70.
9. S. F. Boys and F. Bernardi, *Mol. Phys.*, 1970, **19**, 553.
10. D. R. Herschbach and V. W. Laurie, *J. Chem. Phys.,* 1961, **35**, 458.
11. P. Schwerdtfeger, M. Dolg, W. H. E. Schwarz, G. A. Bowmaker and P. D. W. Boyd, *J. Chem. Phys.,* 1989, **91**, 1762.
12. D. Figgen, G. Rauhut, M. Dolg and H. Stoll, *Chem. Phys.,* 2005, **311**, 227.
13. D. Figgen, K. A. Peterson, M. Dolg and H. Stoll, *J. Chem. Phys.*, 2009, **130**, 164108.
14. M. Gerenkamp and S. Grimme, *Chem. Phys. Lett.*, 2004, *392*, 229.
15. C. Riplinger and F. Neese*, J. Chem. Phys.,* 2013, **138**, 034106.
16. F. Neese, *WIREs Comput. Mol. Sci.,* 2022, **12**, e1606.
17. B. Helmich-Paris, B. de Souza, F. Neese, R. Izsák, *J. Chem. Phys.*, 2021, **155**, 104109.
18. E. D. Glendening, C. R. Landis and F. Weinhold, *WIREs Comput. Mol. Sci.*, 2011, **2**, 1.
19. K. B. Wiberg, *Tetrahedron,* 1968, **24**, 1083.
20. J. Klein, H. Khartabil, J.-C. Boisson, J. Contreras-García, J.-P. Piquemal and E. Hénon, *J. Phys. Chem. A*, 2020, **124**, 1850.
21. E. Matito, J. Poater, M. Solà, M. Duran and P. Salvador, *J. Phys. Chem. A*, 2005, **109**, 9904..
22. R. F. W. Bader, *Chem. Rev.*, 1991, **91**, 893.
23. T. Lu and F. Chen, *J. Comput. Chem.*, 2011, 33, 580.
24. J. Echeverría and S. Alvarez, *Chem. Sci.*, 2023, **14**, 11647.
25. B. Cordero, V. Gómez. A. E. Platero-Prats, M. Revés, J. Echeverría, E. Cremades, F. Barragán and S. Alvarez, *Dalton Trans.***,** 2008, 2832.
26. S. Alvarez, *Dalton Trans***.**, 2013, **42**, 8617.
27. E. D. Glendening and A. Streitwieser, *J. Chem. Phys.*, 1994, **100**, 2900.
28. E. D. Glendening and *J. Phys. Chem. A,* 2005, **109**, 11936.
29. E. D. Glendening, J, K. Badenhoop, A. E. Reed, J. E. Carpenter, J. A. Bohmann, C. M. Morales, P. Karafiloglou and C. R. Landis, F. Weinhold, *NBO 7.0.*, Theoretical Chemistry Institute, University of Wisconsin, Madison, 2018.
30. P. Hohenberg and W. Kohn, *Phys. Rev.,* 1964, **136**, B864.
31. W. Kohn and L. J. Sham, *Phys. Rev.*, 1965, **140**, A1133.
32. M. Ernzerhof and G. E. Scuseria, *J. Chem. Phys.*, 1999, **110**, 5029.
33. S. Grimme, *J. Comput. Chem.,* 2006, **27**, 1787.
34. S. Grimme, J. Antony, S. Ehrlich and H. Krieg, *J. Chem. Phys.*, 2010, **132**, 154104.
35. T. Lu and Q. Chen, *Chemistry–Methods*, 2021, **1**, 231.
36. T. Lu and Q. Chen, *J. Comput. Chem*., 2022, **43**, 539.
37. W. Humphrey, A. Dalke and K. Schulten, *J. Mol. Graph.*, 1996, **14**, 3.

Optimized Structures

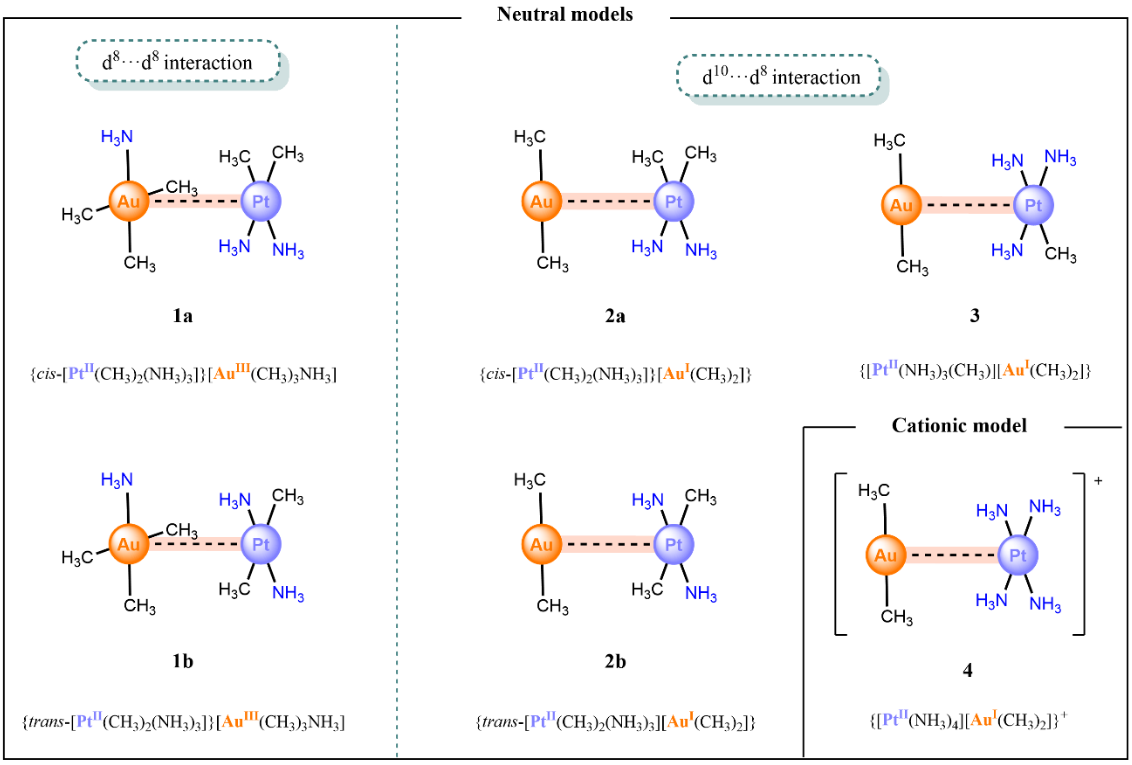

Scheme S1 Theoretical models proposed for study gold-platinum metallophilic interactions.

Table S1 Selected bond lengths (in angstrom) and angles (in degrees) for the MP2 and RHF optimized structures.

Model	**Optimized Structures**				
	$D_{Au\cdots Pt}$	$D_{Au\cdots H\text{-}X\ (X\ =\ N,\ C)}$	$D_{Pt\cdots H\text{-}N}$	$A_{N\text{-}Pt\text{-}Au}$	$A_{L\text{-}Au\text{-}L}$
**1a**^{MP2}	3.5125	2.8788	2.2755	74.49	178.47
**1a**^{RHF}	4.2503	3.5098	2.7014	74.10	179.87
**1b**^{MP2}	3.3671	3.1535	2.5340	85.22	179.43
**1b**^{RHF}	4.8985	3.4326	3.0245	62.77	179.04
**2a**^{MP2}	3.3266	2.7565	2.4037	74.53	178.41
**2a**^{RHF}	4.1506	3.2707	2.7659	71.09	178.40
**2b**^{MP2}	4.2200	2.3063	2.2862	50.28	178.82
**2b**^{RHF}	4.7920	2.9190	3.0775	51.82	178.85
**2c**^{MP2}	3.4000	2.4182	2.6113	68.07	178.42
**3**^{MP2}	3.2823	2.2400	3.4418	75.19	177.32
**3**^{RHF}	4.6197	2.5361	-	46.74	179.76
**4**^{MP2}	2.8779	2.8830	3.5088	87.66	174.28
**4**^{RHF}	3.1546	3.1411	3.6846	87.59	178.31

Potential Energy Curves

Table S2 Absolute energies (kJ·mol^{-1}) as function of Au^{III}-Pt^{II} distance (Å) of the model **1a** calculated at the RHF/def2-TZVP and MP2/def2-TZVP levels of theory with the counterpoise correction for the basis set superposition error and different pseudopotentials (QR = Quasi-Relativistic; NR = Non-Relativistic; FR = Fully-Relativistic).

R	1a QR		1a NR		1a FR	
	ΔE_{int}^{HF}	ΔE_{int}^{MP2}	ΔE_{int}^{HF}	ΔE_{int}^{MP2}	ΔE_{int}^{HF}	ΔE_{int}^{MP2}
2.80	240.355	79.043	256.495	102.384	239.384	78.004
3.00	114.345	-10.378	127.217	6.846	113.681	-11.080
3.20	40.156	-56.409	50.885	-43.261	39.689	-56.899
3.40	-1.910	-76.936	7.323	-66.557	-2.252	-77.296
3.60	-24.298	-82.877	-16.172	-74.417	-24.564	-83.159
3.80	-34.861	-80.879	-27.614	-73.795	-35.082	-81.116
4.00	-38.513	-74.918	-32.021	-68.869	-38.705	-75.127
4.20	-38.297	-67.304	-32.491	-62.076	-38.471	-67.495
4.40	-36.070	-59.335	-30.893	-54.781	-36.228	-59.510

Table S3 Absolute energies (kJ·mol^{-1}) as function of Au^{III}-Pt^{II} distance (Å) of the model **1b** calculated at the RHF/def2-TZVP and MP2/def2-TZVP levels of theory with the counterpoise correction for the basis set superposition error and different pseudopotentials (QR = Quasi-Relativistic; NR = Non-Relativistic; FR = Fully-Relativistic).

R	1b QR		1b NR		1b FR	
	ΔE_{int}^{HF}	ΔE_{int}^{MP2}	ΔE_{int}^{HF}	ΔE_{int}^{MP2}	ΔE_{int}^{HF}	ΔE_{int}^{MP2}
2.80	216.257	65.797	220.960	77.892	215.914	65.343
3.00	93.103	-19.103	95.952	-11.607	92.903	-19.373
3.20	30.499	-53.730	32.457	-48.904	30.374	-53.895
3.40	-0.039	-63.872	1.527	-60.578	-0.121	-63.978
3.60	-13.815	-62.717	-12.403	-60.303	-13.873	-62.789
3.80	-19.006	-56.887	-17.651	-54.984	-19.051	-56.941
4.00	-19.933	-49.583	-18.612	-47.989	-19.970	-49.627
4.20	-18.860	-42.281	-17.582	-40.890	-18.894	-42.321
4.40	-16.964	-35.614	-15.744	-34.372	-16.995	-35.652

Table S4 Absolute energies (kJ·mol^{-1}) as function of Au^{I}-Pt^{II} distance (Å) of the model **2a** calculated at the RHF/def2-TZVP and MP2/def2-TZVP levels of theory with the counterpoise correction for the basis set superposition error and different pseudopotentials (QR = Quasi-Relativistic; NR = Non-Relativistic; FR = Fully-Relativistic).

R	2a QR		2a NR		2a FR	
	ΔE_{int}^{HF}	ΔE_{int}^{MP2}	ΔE_{int}^{HF}	ΔE_{int}^{MP2}	ΔE_{int}^{HF}	ΔE_{int}^{MP2}
2.40	361.290	197.374	382.746	243.518	359.514	195.271
2.60	188.935	59.626	201.380	89.663	187.887	58.309
2.80	89.056	-11.327	96.898	8.521	88.413	-12.170
3.00	30.050	-47.256	35.637	-33.778	29.626	-47.821
3.20	-4.613	-64.079	-0.170	-54.648	-4.916	-64.481
3.40	-24.241	-70.083	-20.443	-63.302	-24.476	-70.389
3.60	-34.458	-69.923	-31.081	-64.929	-34.654	-70.173
3.80	-38.810	-66.372	-35.763	-62.617	-38.985	-66.588
4.00	-39.591	-61.120	-36.841	-58.242	-39.751	-61.313
4.20	-38.292	-55.188	-35.825	-52.944	-38.441	-55.362
4.40	-35.878	-49.185	-33.677	-47.404	-36.015	-49.344

Table S5 Absolute energies (kJ·mol^{-1}) as function of Au^{I}-Pt^{II} distance (Å) of the model **2c** calculated at the RHF/def2-TZVP and MP2/def2-TZVP levels of theory with the counterpoise correction for the basis set superposition error and different pseudopotentials (QR = Quasi-Relativistic; NR = Non-Relativistic; FR = Fully-Relativistic).

R	2c QR		2c NR		2c FR	
	ΔE_{int}^{HF}	ΔE_{int}^{MP2}	ΔE_{int}^{HF}	ΔE_{int}^{MP2}	ΔE_{int}^{HF}	ΔE_{int}^{MP2}
2.40	397.202	226.458	416.664	267.999	395.933	224.841
2.60	212.453	79.913	222.616	105.543	211.824	78.996
2.80	110.219	8.685	115.529	24.392	109.912	8.163
3.00	52.987	-24.401	55.900	-14.702	52.833	-24.707
3.20	20.975	-38.101	22.717	-32.031	20.893	-38.287
3.40	3.364	-41.940	4.532	-38.089	3.319	-42.059
3.60	-5.952	-40.895	-5.063	-38.418	-5.979	-40.975
3.80	-10.491	-37.618	-9.738	-35.998	-10.510	-37.675
4.00	-12.303	-33.507	-11.622	-32.426	-12.318	-33.552
4.20	-12.590	-29.279	-11.956	-28.536	-12.603	-29.316
4.40	-12.064	-25.285	-11.468	-24.755	-12.077	-25.317

Table S6 Absolute energies (kJ·mol^{-1}) as function of Au^{I}-Pt^{II} distance (Å) of the model **3** calculated at the RHF/def2-TZVP and MP2/def2-TZVP levels of theory with the counterpoise correction for the basis set superposition error and different pseudopotentials (QR = Quasi-Relativistic; NR = Non-Relativistic; FR = Fully-Relativistic).

R	3 QR		3 NR		3 FR	
	ΔE_{int}^{HF}	ΔE_{int}^{MP2}	ΔE_{int}^{HF}	ΔE_{int}^{MP2}	ΔE_{int}^{HF}	ΔE_{int}^{MP2}
2.40	309.773	94.786	336.277	147.144	308.557	93.311
2.60	-6.956	-173.300	13.954	-133.796	-7.549	-174.102
2.80	-178.569	-306.042	-160.882	-275.432	-178.850	-306.469
3.00	-270.732	-368.117	-255.282	-343.992	-270.871	-368.346
3.20	-318.262	-392.794	-304.758	-373.748	-318.341	-392.925
3.40	-340.216	-397.450	-328.568	-382.565	-340.275	-397.539
3.60	-347.336	-391.439	-337.451	-380.003	-347.398	-391.517
3.80	-345.835	-379.928	-337.573	-371.334	-345.909	-380.010
4.00	-339.391	-365.815	-332.585	-359.539	-339.480	-365.907
4.20	-330.227	-350.746	-324.691	-346.334	-330.329	-350.848
4.40	-319.697	-335.649	-315.237	-332.704	-319.807	-335.758

Table S7 Absolute energies (kJ·mol^{-1}) as function of Au^{I}-Pt^{II} distance (Å) of the model **4** calculated at the RHF/def2-TZVP and MP2/def2-TZVP levels of theory with the counterpoise correction for the basis set superposition error and different pseudopotentials (QR = Quasi-Relativistic; NR = Non-Relativistic; FR = Fully-Relativistic).

R	4 QR		4 NR		4 FR	
	ΔE_{int}^{HF}	ΔE_{int}^{MP2}	ΔE_{int}^{HF}	ΔE_{int}^{MP2}	ΔE_{int}^{HF}	ΔE_{int}^{MP2}
2.40	-438.988	-604.240	-402.048	-547.106	-440.385	-606.009
2.60	-591.710	-720.525	-566.805	-682.718	-592.471	-721.596
2.80	-662.948	-762.356	-645.989	-737.963	-663.388	-763.036
3.00	-690.532	-767.240	-679.030	-752.205	-690.816	-767.701
3.20	-694.372	-754.068	-686.811	-745.691	-694.574	-754.401
3.40	-685.265	-732.334	-680.651	-728.805	-685.420	-732.589
3.60	-669.211	-706.847	-666.838	-706.917	-669.339	-707.053
3.80	-649.610	-680.080	-648.956	-682.866	-649.724	-680.259
4.00	-628.428	-653.346	-629.099	-658.199	-628.538	-653.510
4.20	-606.822	-627.371	-608.506	-633.790	-606.932	-627.528
4.40	-585.465	-602.551	-587.905	-610.130	-585.577	-602.705

Table S8 Absolute energies (kJ·mol^{-1}) as function of Pt^{II}-H distance (Å) of the models **2b** calculated at the RHF/def2-TZVP and MP2/def2-TZVP levels of theory with the counterpoise correction for the basis set superposition error.

R	2b QR	
	ΔE_{int}^{HF}	ΔE_{int}^{MP2}
1.60	272.749	141.560
1.80	120.504	18.960
2.00	43.632	-33.558
2.20	5.037	-53.318
2.40	-13.473	-57.668
2.60	-21.306	-54.984
2.80	-23.550	-49.434
3.00	-23.008	-43.078
3.20	-21.212	-36.896
3.40	-18.970	-31.309
3.60	-16.685	-26.454

Table S9 Absolute energies (kJ·mol^{-1}) as function of Au^{III}-Au^{III} distance (Å) of the model **S1** calculated at the RHF/def2-TZVP and MP2/def2-TZVP levels of theory with the counterpoise correction for the basis set superposition error and different pseudopotentials (QR = Quasi-Relativistic; NR = Non-Relativistic; FR = Fully-Relativistic).

**R**	**S1 QR**		**S1 NR**		**S1 FR**	
	ΔE_{int}^{HF}	ΔE_{int}^{MP2}	ΔE_{int}^{HF}	ΔE_{int}^{MP2}	ΔE_{int}^{HF}	ΔE_{int}^{MP2}
2.80	179.287	38.033	191.281	53.671	179.203	38.005
3.00	85.718	-21.562	94.486	-11.133	85.638	-21.593
3.20	34.173	-47.647	40.870	-40.435	34.093	-47.686
3.40	6.732	-56.029	12.029	-50.847	6.655	-56.075
3.60	-7.086	-55.533	-2.764	-51.652	-7.158	-55.581
3.80	-13.355	-51.005	-9.732	-47.972	-13.420	-51.052
4.00	-15.550	-45.012	-12.448	-42.548	-15.609	-45.059
4.20	-15.636	-38.851	-12.942	-36.783	-15.690	-38.895
4.40	-14.710	-33.125	-12.346	-31.347	-14.758	-33.167

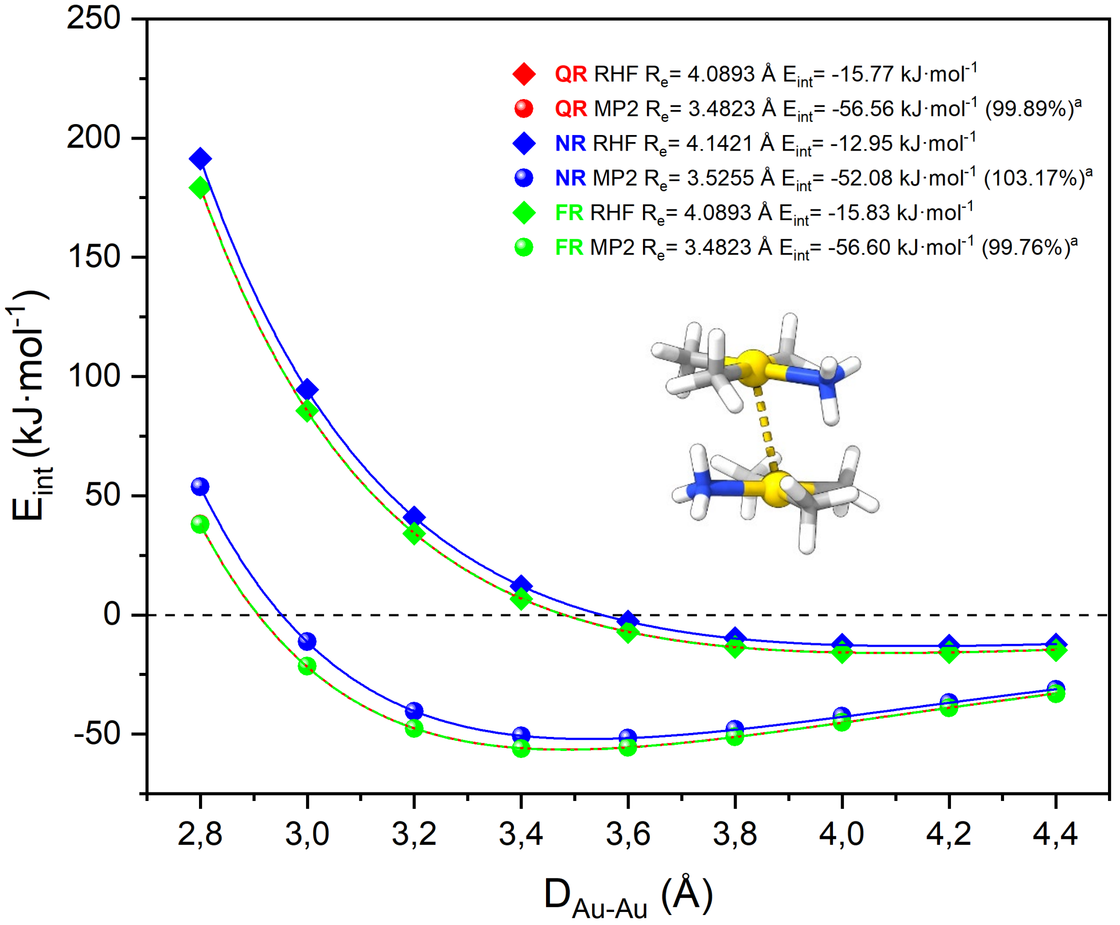

Figure S1 The total interaction energy as a function of the Au^{III}-Au^{III} distance for model **S1**, calculated at the RHF/def2-TZVP and MP2/def2-TZVP levels of theory with different relativistic pseudopotentials (QR Quasi-Relativistic; NR Non-Relativistic; FR Fully-Relativistic). Raw data are provided in **Table S9** The dispersion contribution was calculated with equation **S1**.

Effective charges

Table S10 NBO effective charges calculated at the MP2/def2-TVZP level of theory for the models **1**–**4**. The charges of the atoms in their corresponding isolated monomer are shown in parentheses.

Atoms	1a	1b	2a	2b	2c	3	4
C (1)	-1.00 (-1.00)	-1.00 (-1.00)	-1.01 (-0.98)	-0.99 (-0.98)	-0.98 (-1.00)	-1.10 (-1.11)	-1.12 (-1.11)
C (2)	-0.80 (-0.77)	-0.77 (-0.77)	-	-	-	-1.13 (-1.11)	-1.12 (-1.11)
C (3)	-1.00 (-1.00)	-1.01 (-1.00)	-	-	-	-	-
N (1)	-1.00 (-0.98)	-1.00 (-0.98)	-1.03 (-1.02)	-1.04 (-1.02)	-1.03 (-1.02)	-	
C (4) / N (1)	-0.84 (-0.83)	-1.03 (-0.99)	-0.84 (-0.83)	-0.99 (-1.00)	-0.96 (-1.00)	-0.81 (-0.80)	-0.92 (-0.91)
C (5) / N (2)	-0.84 (-0.83)	-0.96 (-1.00)	-0.84 (-0.83)	-0.98 (-1.00)	-1.03 (-1.00)	-0.91 (-0.91)	-0.92 (-0.90)
N (2) / N (3)	-0.96 (-0.97)	-0.88 (-0.88)	-0.96 (-0.97)	-0.89 (-0.88)	-0.88 (-0.88)	-1.01 (-0.99)	-0.92 (-0.91)
N (3) / N (4)	-0.96 (-0.97)	-0.88 (-0.88)	-0.96 (-0.97)	-0.88 (-0.88)	-0.88 (-0.88)	-0.91 (-0.91)	-0.92 (-0.90)

Table S11 QTAIM effective charges calculated at the MP2/def2-TVZP level of theory for the models **1**–**4**. The charges of the atoms in their corresponding isolated monomer are shown in parentheses.

Atoms	1a	1b	2a	2b	2c	3	4
C (1)	-0.33 (-0.33)	-0.31 (-0.33)	-0.27 (-0.26)	-0.27 (-0.26)	-0.28 (-0.26)	-0.33 (-0.35)	-0.39 (-0.35)
C (2)	-0.19 (-0.18)	-0.19 (-0.18)	-	-	-	-0.35 (-0.35)	-0.39 (-0.35)
C (3)	-0.33 (-0.34)	-0.29 (-0.34)	-	-	-	-	-
N (1)	-1.18 (-1.16)	-1.18 (-1.16)	-1.17 (-1.15)	-1.15 (-1.15)	-1.17 (-1.15)	-	-
C (4) / N (1)	-0.23 (-0.24)	-0.34 (-0.32)	-0.23 (-0.24)	-0.33 (-0.32)	-0.31 (-0.32)	-0.21 (-0.24)	-1.15 (-1.14)
C (5) / N (2)	-0.24 (-0.24)	-0.32 (-0.32)	-0.23 (-0.24)	-0.35 (-0.32)	-0.34 (-0.32)	-1.13 (-1.12)	-1.13 (-1.14)
N (2) / N (3)	-1.15 (-1.14)	-1.12 (-1.11)	-1.12 (-1.14)	-1.10 (-1.11)	-0.10 (-1.11)	-1.19 (-1.15)	-1.15 (-1.14)
N (3) / N (4)	-1.15 (-1.14)	-1.11 (-1.11)	-1.12 (-1.14)	-1.07 (-1.11)	-1.10 (-1.11)	-1.12 (-1.13)	-1.13 (-1.14)

Bond orders

Table S12 Calculated bond order indexes of the additional BCPs in the model **1a** calculated at the MP2/def2-TZVP level of theory. Wiberg Bond Index (WBI) and Intrinsic Bond Strength Index (IBSI).

			**1a**		
BCP	**$Pt^{II}\cdots$H-N**	**C⋯H-N**	**C⋯H-N**	**C-H⋯H-C**	**C-H⋯H-C**
**WBI**	0.04	0.00	0.00	0.00	0.00
**IBSI**	0.04	0.01	0.01	0.00	0.00

Table S13: Calculated bond order indexes of the additional BCPs in the model **1b** calculated at the MP2/def2-TZVP level of theory. Wiberg Bond Index (WBI) and Intrinsic Bond Strength Index (IBSI).

		**1b**		
BCP	**$Pt^{II}\cdots$H-N**	**C⋯H-N**	**C⋯H-N**	**C-H⋯H-C**
**WBI**	0.02	0.00	0.00	0.00
**IBSI**	0.03	0.01	0.01	0.06

Table S14: Calculated bond order indexes of the additional BCPs in the models **2a** calculated at the MP2/def2-TZVP level of theory. Wiberg Bond Index (WBI) and Intrinsic Bond Strength Index (IBSI).

		**2a**	
BCP	**$Pt^{II}\cdots$H-N**	**$Au^{I}\cdots$H-N**	**$Au^{I}\cdots$H-N**
**WBI**	0.03	0.01	0.01
**IBSI**	0.03	0.01	0.01

Table S15 Calculated bond order indexes of the additional BCPs in the models **2c** calculated at the MP2/def2-TZVP level of theory. Wiberg Bond Index (WBI) and Intrinsic Bond Strength Index (IBSI).

	**2c**			
BCP	**Au^{I}···H-N**	**C···H-N**	**Pt^{II}···H-N**	**C-H···H-C**
**WBI**	0.01	0.00	0.01	0.00
**IBSI**	0.02	0.01	0.01	0.00

Table S16 Calculated bond order indexes of the additional BCPs in the models **3** calculated at the MP2/def2-TZVP level of theory. Wiberg Bond Index (WBI) and Intrinsic Bond Strength Index (IBSI).

		**4**	
BCP	**Au···H-N**	**C···H-N**	**C···H-N**
**WBI**	0.05	0.01	0.00
**IBSI**	0.04	0.02	0.00

NEDA

Table S17 NEDA contributions to the total interaction energy between the monomers at the potential energy MP2 curve minimum for the models **1**–**4**, calculated at the PBE0-D3(BJ)/def2-TZVP level of theory.[a]

Model	ΔE_{ES} (%)[a]	ΔE_{XC} (%)[a]	ΔE_{POL} (%)[a]	ΔE_{CT} (%)[a]	ΔE_{SE}	ΔE_{DEF}	ΔE_{EL}	ΔE_{CORE}	ΔE_{int}
**1a**	-104.5 (29.5)	-93.1 (26.3)	-54.1 (15,3)	-102.8 (29.0)	27.1	261.1	-131.5	140.8	-93.4
**1b**	-87.7 (25.2)	-102.9 (29.5)	-65.3 (18.7)	-92.6 (26.6)	32.6	274.8	-120.3	139.2	-73.7
**2a**	-104.4 (34.1)	-80.8 (26.4)	-41.3 (13.5)	-80.0 (26.1)	21.0	224.8	-124.7	123.1	-81.6
**2b**	-75.9 (31.1)	-53.3 (21.8)	-7.5 (3.0)	-108.1 (44.2)	3.0	177.4	-80.3	121.1	-67.3
**2c**	-65.1 (26.0)	-78.2 (31.2)	-43.2 (17.2)	-64.2 (25.2)	21.8	200.7	-86.5	100.7	-50.0
**3**	-341.0 (60.1)	-82.9 (14.6)	-78.0 (13.8)	-65.0 (11.5)	39.3	220.4	-379.6	98.2	-346.5
**4**	-834.1 (63.6)	-152.6 (11.6)	-145.3 (11.1)	-178.9 (13.6)	75.8	529.4	-903.6	301.0	-781.5

[a] The percentages contribution are calculated with respect to the sum of the stabilizing contributions ($\Delta E_{ES,}$ $\Delta E_{XC,}$ $\Delta E_{POL,}$ ΔE_{CT}), equation **S6**.

IGMH analysis

Table S18 Results of IGMH analyses for the metal and hydrogen atoms in models **1**–**4** calculated at the MP2/def2-TZVP level of theory. Values of IGMH atomic indexes (δg^{atom}) and intrinsic bond strength indexes for weak interactions (IBSIW) are in au.

Model	**1a**		**1b**		**2a**		**2b**		**2c**		**3**		**4**	
Atom	Au^{III}	Pt^{II}	Au^{III}	Pt^{II}	Au^{I}	Pt^{II}	Pt^{II}	H	Au^{I}	Pt^{II}	Au^{I}	Pt^{II}	Au^{I}	Pt^{II}
**δg^{atom}** (a.u.)	0.29	0.33	0.39	0.33	0.39	0.31	0.20	0.31	0.38	0.27	0.56	0.26	0.76	0.46
**δg^{atom}** (%)	19.58	22.06	24.83	20.58	35.13	28.18	23.32	35.96	37.68	26.53	40.13	18.51	40.76	24.79
**IBSW** (a.u.)	2.35	3.71	2.46	3.53	3.35	3.12	2.43	4.32	3.25	2.28	6.21	2.02	7.78	4.52

Table S19 Results of IGMH analyses for the additional BCPs in models **1a** calculated at the MP2/def2-TZVP level of theory. Values of IGMH atomic pair indexes (δg^{pair}) and intrinsic bond strength indexes for weak interactions (IBSIW) are in au.

	**1a**				
BCP	**Pt^{II}···H-N**	**C···H-N**	**C···H-N**	**C-H···H-C**	**C-H···H-C**
**δg^{pair}** (a.u./Å^2)	0.10	0.04	0.04	0.03	0.03
**δg^{pair}** (%)	7.01	2.94	2.94	1.67	1.67
**IBSIW** (a.u.)	1.85	0.60	0.59	0.42	0.42

Table S20 Results of IGMH analyses for the additional BCPs in models **1b** calculated at the MP2/def2-TZVP level of theory. Values of IGMH atomic pair indexes (δg^{pair}) and intrinsic bond strength indexes for weak interactions (IBSIW) are in au.

	**1b**			
BCP	**Pt^{II}···H-N**	**C···H-N**	**C···H-N**	**C-H···H-C**
**δg^{pair}** (a.u./Å^2)	0.09	0.06	0.04	0.02
**δg^{pair}** (%)	5.73	3.73	2.28	1.19
**IBSIW** (a.u.)	1.34	1.11	0.59	0.30

Table S21 Results of IGMH analyses for the additional BCPs in models **2a** calculated at the MP2/def2-TZVP level of theory. Values of IGMH atomic pair indexes (δg^{pair}) and intrinsic bond strength indexes for weak interactions (IBSIW) are in au.

	**2a**		
BCP	**Pt^{II}···H-N**	**Au^{I}···H-N**	**Au^{I}···H-N**
**δg^{pair}** (a.u./Å^2)	0.086	0.054	0.054
**δg^{pair}** (%)	7.86	4.89	4.89
**IBSIW** (a.u.)	1.37	0.66	0.66

Table S22 Results of IGMH analyses for the additional BCPs in models **2c** calculated at the MP2/def2-TZVP level of theory. Values of IGMH atomic pair indexes (δg^{pair}) and intrinsic bond strength indexes for weak interactions (IBSIW) are in au.

	**2c**			
BCP	**Au^I^···H-N**	**Pt^II^···H-N**	**C···H-N**	**C-H···H-C**
δg^{pair} (a.u.)	0.07	0.06	0.04	0.01
δg^{pair} (%)	6.91	5.58	4.31	1.26
**IBSIW** (a.u.)	0.96	0.67	0.72	0.17

Table S23 Results of IGMH analyses for the additional BCPs in models **3** calculated at the MP2/def2-TZVP level of theory. Values of IGMH atomic pair indexes (δg^{pair}) and intrinsic bond strength indexes for weak interactions (IBSIW) are in au.

	**3**		
BCP	**Au···H-N**	**C···H-N**	**C···H-N**
δg^{pair} (a.u./Å^2)	0.117	0.058	0.025
δg^{pair} (%)	8.42	4.21	1.79
**IBSIW** (a.u.)	2.20	1.11	0.22

Table S24 Results of IGMH analyses for the additional BCPs in models **4** calculated at the MP2/def2-TZVP level of theory. Values of IGMH atomic pair indexes (δg^{pair}) and intrinsic bond strength indexes for weak interactions (IBSIW) are in au.

	**4**			
BCP	**C···H-N**	**C···H-N**	**N-H···H-N**	**N-H···H-C**
δg^{pair} (a.u./Å^2)	0.056	0.056	0.04	0.04
δg^{pair} (%)	3.01	3.01	2.09	2.09
**IBSIW** (a.u.)	1.08	1.08	0.99	0.99

IRI analysis

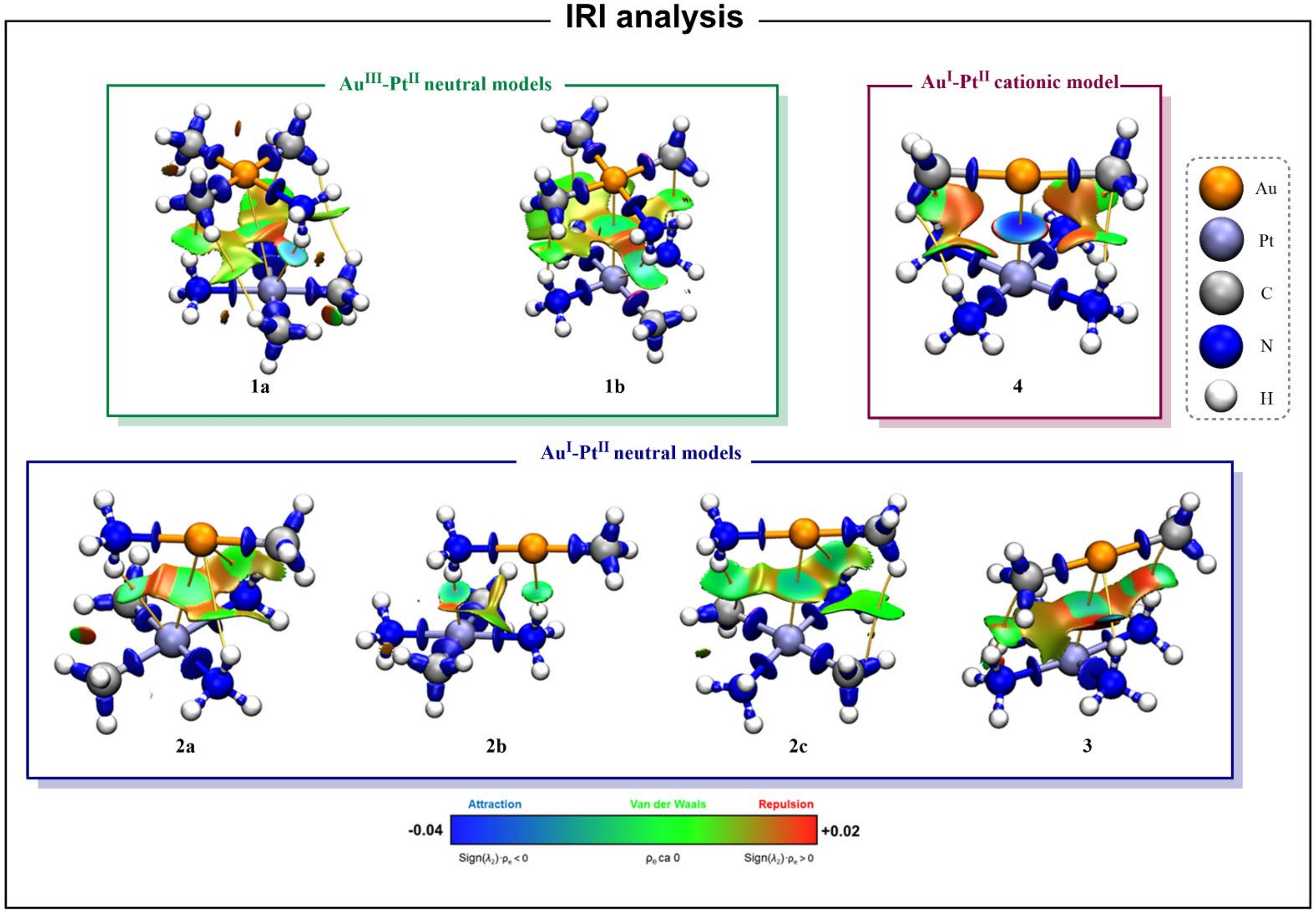

Figure S2 The QTAIM (3,-1) BCPs (orange dots), (3,+1) RCP (yellow dots), bond paths (yellow strings) and the IGMH isosurface (isovalues = 1.1 for models **1a**, **1b**, **2b** and **2c**; isovalues = 1 for models **2a**, **3** and **4**) are superimposed for models **1** - **4**. The RGB colour scale refers to the IRI isosurface.

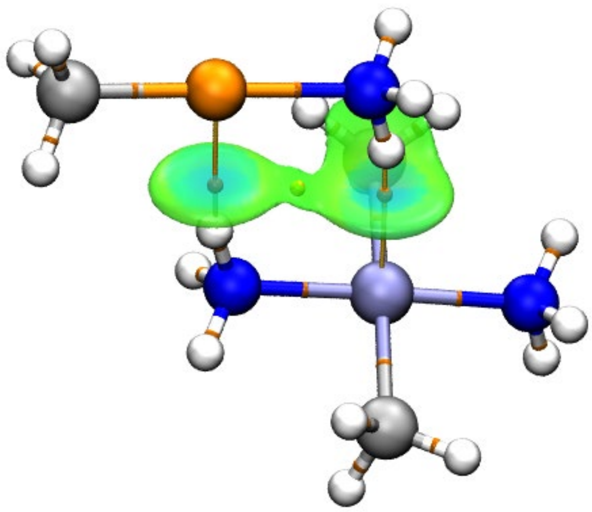

Figure S3 The QTAIM (3, -1) BCPs (orange dots), (3, +1) RCPs (yellow dots), bond paths (yellow strings) and the IGMH isosurface (isovalue = 1.0) of model **2b**.

Cartesian coordinates

Model 1a (MP2/def2-TZVP optimization)

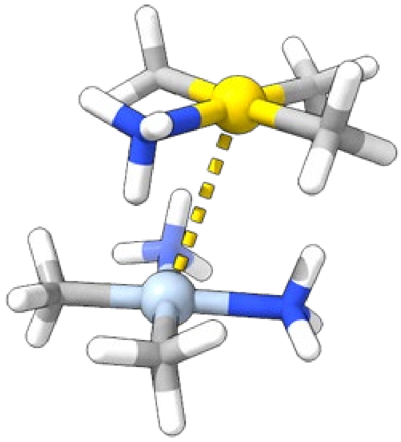

Au	-0.23463900	-0.19330300	1.73220800
H	1.26629600	-2.30451500	-1.68020100
H	0.35124900	-2.10973300	-0.33460600
H	-0.35099400	-2.53494000	-1.76306900
C	2.23865300	0.12317500	-2.02457900
H	2.44912800	-0.28890900	-3.02092000
H	2.72596300	1.10202100	-1.97925300
H	2.74486300	-0.52256100	-1.29095700
H	0.98431700	2.20924900	1.39352600
H	2.16334300	1.04750000	1.44496800
H	1.16746300	1.15790600	0.10705900
C	-1.71430200	1.27410900	1.72427500
H	-2.72050600	0.88623900	1.53518600
H	-1.74101600	1.73889500	2.71581900
H	-1.50140800	2.06023700	0.99085300
C	-1.61355600	-1.57028800	2.23875800
H	-2.01613500	-2.01506000	1.32286400
H	-1.16046700	-2.35771400	2.84078600
H	-2.43016000	-1.10444900	2.79006900
C	1.25225100	-1.64855300	1.85067700
H	2.06569300	-1.45495600	1.14212600
H	1.67480200	-1.62500600	2.86102600
H	0.88703900	-2.66803500	1.68975500
C	0.21361500	2.12072100	-2.11165100
H	-0.15886100	2.28225900	-3.13242800
H	-0.46873600	2.64861600	-1.42815900
H	1.18349000	2.62353200	-2.04705200
N	0.37157800	-1.95587000	-1.34277800
N	1.21477000	1.25167000	1.14136000
Pt	0.26266200	0.16008300	-1.72688200

N	-1.86924400	0.25492400	-1.43924200
H	-2.21436300	1.12826000	-1.83180400
H	-2.42028500	-0.49434500	-1.85036500
H	-2.06633900	0.27779100	-0.43867700

Model 1a (RHF/def2-TZVP optimization)

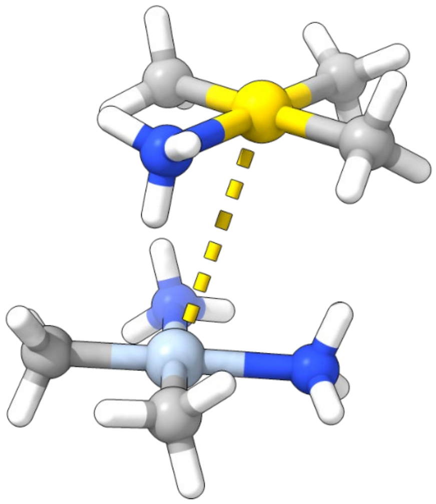

Au	-0.31197200	-0.25575600	2.08949700
H	1.00154400	-2.41632900	-2.28323700
H	0.79148100	-2.10704500	-0.71136400
H	-0.48015500	-2.47496700	-1.63669300
C	2.39022000	0.15622500	-2.33464800
H	2.65230200	0.37218600	-3.36864600
H	2.91165500	0.89392700	-1.72727300
H	2.82959800	-0.81370300	-2.09143900
H	0.87466400	2.17545300	1.57294200
H	2.06026100	1.06772000	1.64196800
H	1.13224200	1.18206500	0.31749000
C	-1.88085500	1.17230700	2.00750700
H	-2.82607200	0.73212200	1.70282600
H	-2.03910700	1.60497200	2.99226300
H	-1.65803100	1.99141700	1.32553000
C	-1.63769900	-1.63597900	2.79178200
H	-2.07527300	-2.15793200	1.94785700
H	-1.13324100	-2.35384100	3.42166300
H	-2.42607500	-1.14943400	3.34711100
C	1.22379800	-1.71646300	2.19279500
H	2.06701000	-1.47382600	1.54785400
H	1.60323400	-1.78489200	3.20930100
H	0.87018500	-2.70687600	1.92152200
C	0.33884100	2.22782900	-2.50448600
H	-0.11360000	2.40444100	-3.48219100
H	-0.25151500	2.79709200	-1.78107300
H	1.32492100	2.67936700	-2.53276900

N	0.39030000	-1.98258400	-1.62063000
N	1.12845800	1.24099000	1.32218200
Pt	0.35240100	0.21647200	-2.08193800
N	-1.87835700	0.35823800	-1.81234700
H	-2.16337200	1.20432300	-2.26399300
H	-2.41935900	-0.38910600	-2.19888100
H	-2.11226900	0.44053700	-0.84179300

Model 1b (MP2/def2-TZVP optimization)

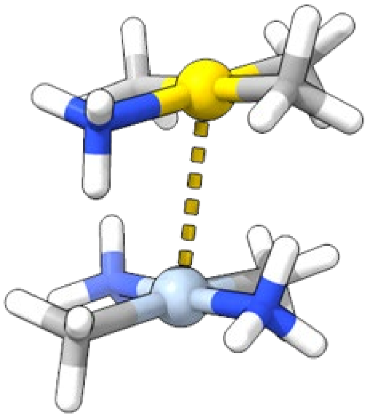

Au	0.00378300	-0.23018200	1.68499100
H	-1.86535200	-1.43981500	-2.19430600
H	-2.01081100	-0.93864500	-0.63541200
H	-2.58096700	0.00885200	-1.86373200
C	0.83127000	-1.65346700	-1.56084800
H	1.09853100	-2.00168200	-2.56967800
H	1.74830900	-1.68857400	-0.95784700
H	0.16688300	-2.41643800	-1.13295700
H	-1.69855800	1.88430900	1.57151900
H	-0.15974700	2.47401300	1.63071600
H	-0.72226200	1.78102000	0.22789200
C	-1.92345600	-1.01976000	1.74864500
H	-1.98474200	-1.98357200	1.23040000
H	-2.20470200	-1.20957400	2.78908000
H	-2.68383000	-0.34687100	1.32969700
C	0.70880000	-2.06936100	2.10745300
H	0.85550800	-2.58759000	1.15835900
H	1.65978100	-1.99532000	2.63471200
H	-0.01319500	-2.61342100	2.71649300
C	1.96371900	0.46549900	1.70675100
H	2.05023300	1.49439300	1.33200800
H	2.36357000	0.46004400	2.72523000
H	2.60837800	-0.18713400	1.10810900
N	-1.82975000	-0.62124800	-1.58933400
N	-0.74457400	1.73225500	1.25599100
Pt	-0.01567400	0.23335400	-1.65002600
C	-0.85531000	2.13530200	-1.94496800
H	-0.28167600	2.98761200	-1.54392000
H	-0.91483300	2.30976900	-3.02849100
H	-1.88271800	2.27872200	-1.57099900
N	1.84117200	0.99886800	-1.65509500
H	1.87131200	1.97486000	-1.94340900

H	2.23095300	0.93128600	-0.71391300
H	2.45041800	0.46655900	-2.27383800

Model 1b (RHF/def2-TZVP optimization)

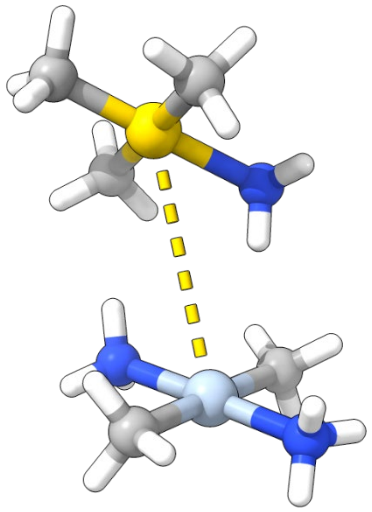

Au	-0.05505900	-0.37465100	2.44563900
H	-1.25344400	-1.90479000	-2.00855400
H	-1.37290000	-1.02638500	-0.65614300
H	-2.31456900	-0.67773400	-1.91743000
C	1.25729800	-1.37890700	-2.90060700
H	0.78746500	-1.99442900	-3.67218700
H	2.25252300	-1.13987400	-3.28213900
H	1.42260300	-2.03464900	-2.04326300
H	-0.90786700	2.11202800	1.61798300
H	0.71438800	2.13050900	1.63839700
H	-0.08399200	1.46102200	0.39487700
C	-2.09855400	-0.72904800	1.96381900
H	-2.24862700	-1.70841100	1.51566500
H	-2.69579300	-0.70911000	2.87114500
H	-2.51644300	0.01788000	1.28954100
C	0.02246200	-2.15935200	3.42381700
H	0.71175600	-2.80927900	2.90024200
H	0.39335500	-1.99541800	4.42631300
H	-0.95273800	-2.62011700	3.46511300
C	1.96771700	-0.02757600	2.95843600
H	2.53849500	0.24832200	2.07293700
H	2.03706200	0.79800600	3.66568800
H	2.45395800	-0.88426700	3.40759900
N	-1.39648100	-0.97896100	-1.65685300
N	-0.08739600	1.58615300	1.39114500
Pt	0.05973600	0.31915200	-2.40209900
C	-1.17407400	2.03362200	-1.95236200
H	-0.62535500	2.89226300	-1.55095500
H	-1.65739100	2.39661000	-2.86200000

H	-1.98723100	1.84921800	-1.24565700
N	1.53078300	1.58031100	-3.18752500
H	1.26835800	2.54441700	-3.14110300
H	2.39346800	1.45700300	-2.69500900
H	1.69695100	1.34050500	-4.14519600

Model 2a (MP2/def2-TZVP optimization)

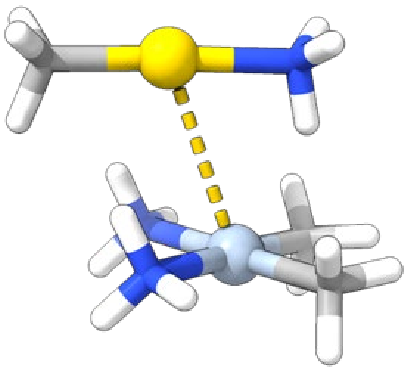

H	2.36803700	-1.24840900	-1.85484800
H	1.45017300	-1.54142100	-0.52310300
H	1.09540400	-2.27217100	-1.95852700
C	1.91490200	1.21142700	-2.61341400
H	2.21681200	0.75115300	-3.56428700
H	1.83952200	2.28783600	-2.79671400
H	2.74430400	1.06266900	-1.90407600
C	-0.82354300	1.99019700	-2.36751700
H	-1.47336300	1.80058100	-3.23293000
H	-1.47817800	2.26347600	-1.52492200
H	-0.22848100	2.87594300	-2.61101900
N	1.41541300	-1.40553200	-1.53334800
Pt	0.23764300	0.34919300	-1.95697900
N	-1.59439500	-0.54959100	-1.26308400
H	-2.36236500	0.09684200	-1.43008500
H	-1.84943800	-1.43470400	-1.69409700
H	-1.52784300	-0.69452000	-0.25569400
Au	0.50367600	0.23823600	1.35712100
N	0.98654100	2.16050800	0.64661900
H	1.94360700	2.45127000	0.82645100
C	0.03634900	-1.60138400	1.97889500
H	-0.81544100	-1.57998000	2.66507700
H	-0.22976800	-2.27766000	1.15706900
H	0.87334700	-2.06024400	2.51343400
H	0.36733600	2.89953700	0.96799200
H	0.86187800	2.04312800	-0.36994800

Model 2a (RHF/def2-TZVP optimization)

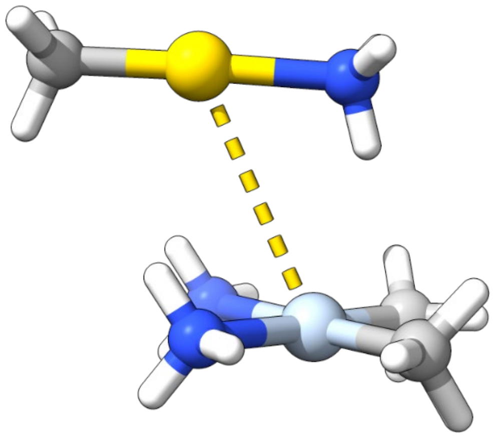

H	2.41213400	-1.23109600	-1.94655100
H	1.40610300	-1.63025400	-0.74376000
H	1.18677700	-2.24357000	-2.22851300
C	1.95810000	1.33659500	-2.95343200
H	1.86821800	1.53677800	-4.01919100
H	2.13696900	2.29891900	-2.47587400
H	2.87066600	0.75048300	-2.82033100
C	-0.85078300	2.07130200	-2.70904600
H	-1.56447400	1.84141700	-3.50267100
H	-1.43923000	2.41691100	-1.85369000
H	-0.26584600	2.91851100	-3.05082100
N	1.45669200	-1.42345000	-1.72266400
Pt	0.26613300	0.40848300	-2.25032400
N	-1.61639300	-0.55894100	-1.48319100
H	-2.35314500	0.10746800	-1.60127700
H	-1.88089500	-1.39162500	-1.97079900
H	-1.55249000	-0.77622400	-0.50737900
Au	0.48809900	0.12467900	1.88460200
N	0.98161300	2.06015300	0.95256800
H	1.92219600	2.34705000	1.13767700
C	0.01441500	-1.71322700	2.70653700
H	-0.84460000	-1.63148800	3.36586600
H	-0.22488500	-2.45766100	1.95017500
H	0.84418400	-2.09906400	3.29120100
H	0.37374800	2.79672400	1.25129800
H	0.87882400	1.95750600	-0.04234800

Model 2b (MP2/def2-TZVP optimization)

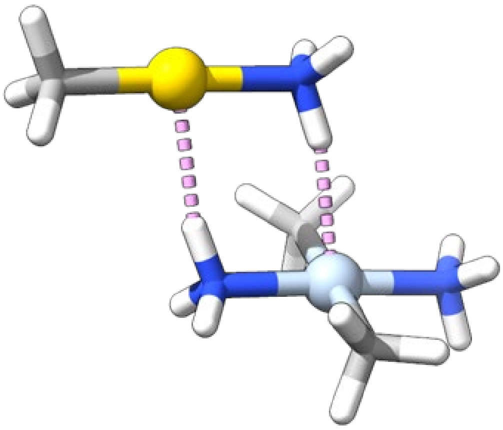

Au	-0.94373400	-0.08408700	2.08870100
H	-0.82017200	-1.71155400	-1.37634100
H	-1.09424700	-0.65006500	-0.14202300
H	-1.88244100	-0.46483200	-1.57357100
C	1.62178800	-1.25970000	-2.20822100
H	1.18732800	-1.85409300	-3.02528600
H	2.66647400	-1.07364200	-2.49654200
H	1.66937400	-1.91881900	-1.33041600
N	-0.98663400	-0.72843600	-1.16588300
Pt	0.49371900	0.44448300	-1.84317200
C	-0.67953200	2.14322200	-1.52274500
H	-0.10482500	3.03241100	-1.21470600
H	-1.20873600	2.43459300	-2.44097700
H	-1.45431400	2.01522700	-0.75334800
N	1.96946500	1.59323700	-2.57903700
H	1.84455500	2.57904900	-2.35668800
H	2.88192300	1.29475000	-2.24022000
H	1.98502600	1.50461000	-3.59428400
C	-2.34676200	-1.34379100	2.73083300
H	-3.34875200	-0.97055200	2.50211700
H	-2.24125500	-2.33310000	2.27682000
H	-2.27537400	-1.46837800	3.81505700
N	0.53218500	1.23382600	1.36931500
H	0.27288900	2.20881600	1.50042300
H	1.43671400	1.08860100	1.81040100
H	0.64372000	1.08558400	0.34618100

Model 2b (RHF/def2-TZVP optimization)

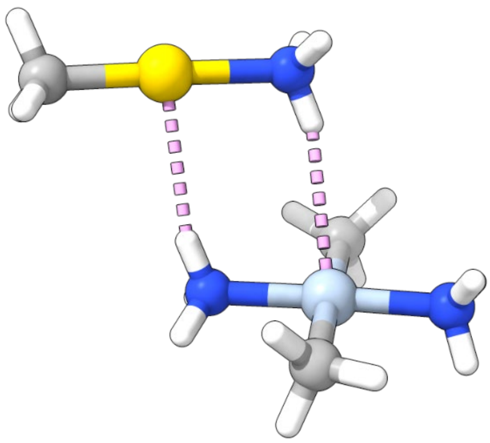

Au	-0.85915800	0.17855600	2.53253300
H	-1.19198300	-1.67801300	-1.55731400
H	-1.06420100	-0.78808300	-0.21415000
H	-2.02794100	-0.29298900	-1.40834300
C	1.37264300	-1.57744500	-2.50221800
H	0.80715600	-2.15661200	-3.23672700
H	2.38515100	-1.50126700	-2.90567700
H	1.45762000	-2.20834400	-1.61501900
N	-1.15406300	-0.73948000	-1.21209800
Pt	0.43783000	0.29749800	-2.07905300
C	-0.52334200	2.19448700	-1.70383200
H	0.15573400	2.98231700	-1.35886900
H	-0.96858000	2.57910800	-2.62394000
H	-1.33803500	2.16889500	-0.97600800
N	2.03354600	1.29368900	-2.99368700
H	1.91161100	2.28647800	-2.99004000
H	2.89456600	1.07248500	-2.53396200
H	2.11420300	0.98576100	-3.94289200
C	-2.06983200	-1.30683000	3.30188000
H	-3.08286200	-1.23158800	2.91736500
H	-1.69102600	-2.29471500	3.05648100
H	-2.12046500	-1.23154800	4.38396300
N	0.42965200	1.75104700	1.66901100
H	0.10544900	2.66579500	1.91270300
H	1.37339800	1.66236100	1.98877500
H	0.43131300	1.68579200	0.66750600

Model 2c (MP2/def2-TZVP optimization, metals freeze)

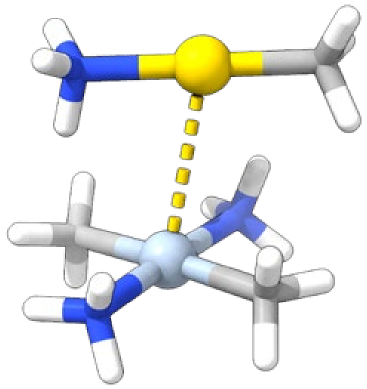

N	3.93149100	1.18435900	1.10946600
H	3.77831300	1.74187700	0.27080600
H	4.58404300	1.67852700	1.71486300
H	4.37840400	0.31758300	0.81414000
C	1.53027000	0.03396800	0.17801600
H	1.41721200	0.84752500	-0.55154600
H	2.22209700	-0.69973200	-0.26168100
H	0.55206300	-0.46390600	0.21710700
C	2.90825800	1.38214600	3.87316200
H	2.14220500	1.79647800	4.54828600
H	3.34095400	0.52503400	4.40815300
H	3.71258900	2.13807800	3.84977000
N	0.43697600	0.22591800	2.83971200
H	0.47938000	0.15977100	3.85471700
H	-0.24426800	0.95241600	2.59133100
H	0.09682500	-0.65993300	2.47026200
Pt	2.18937400	0.74170200	2.00931200
Au	-0.16562500	3.16132500	1.61045300
C	-1.70445600	2.29285000	0.68696800
H	-2.17506600	2.99042500	-0.01120900
H	-1.36686700	1.42307700	0.11528500
H	-2.47786800	1.96520500	1.38879000
N	1.49036100	4.02921100	2.59135600
H	1.25950000	4.48680500	3.46880100
H	2.10377300	3.23156500	2.79181400
H	1.99321600	4.69832600	2.01563100

Model 3 (MP2/def2-TZVP optimization)

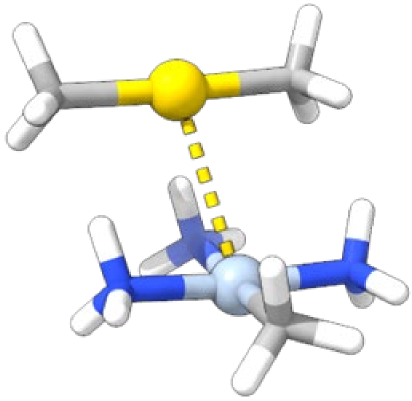

H	0.09900800	-2.44251300	-0.95968100
H	-0.00424800	-1.22971500	0.18171500
H	-1.35941200	-1.86128300	-0.51946200
C	0.27564700	1.77489900	-3.07072800
H	-0.62905400	2.24685400	-3.47120800
H	0.78665300	2.51975200	-2.44889200
H	0.90886000	1.54969100	-3.93665600
N	-0.39677600	-1.59080700	-0.70734600
Pt	-0.08129500	0.14383000	-1.98470000
N	-2.02347900	0.66773200	-1.83864400
H	-2.24298400	1.45633600	-2.44457800
H	-2.68229900	-0.07444000	-2.06485900
H	-2.18745800	0.96484500	-0.87108800
Au	0.88085800	0.60167900	1.11981800
C	2.87059400	0.05117000	1.05557200
H	3.26955800	0.09169200	2.07513100
H	3.07469100	-0.97204600	0.70714000
H	3.50805300	0.72468000	0.46479800
N	1.90441900	-0.20218800	-2.00426200
H	2.18453600	-1.13081200	-2.31333800
H	2.40240600	0.47007300	-2.58532200
H	2.22005200	-0.07080100	-1.03022100
C	-1.09428600	1.17397100	1.27686200
H	-1.81092800	0.37152200	1.03826200
H	-1.35095900	1.49850200	2.29138800
H	-1.30744600	2.03003500	0.62010200

Model 3 (MP2/def2-TZVP optimization)

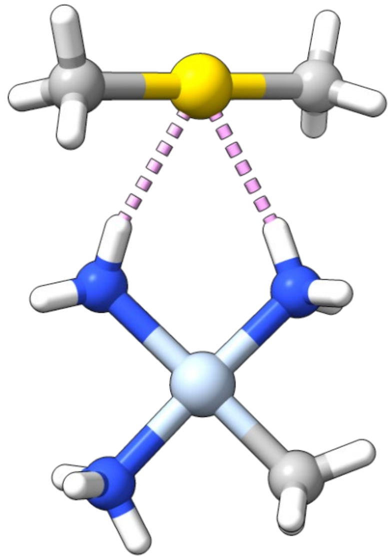

H	1.89461000	-1.51560200	-0.92159200
H	0.82406700	-0.80480500	0.03226100
H	0.33030400	-1.86406300	-1.06232900
C	0.38111300	2.05269200	-3.73845100
H	-0.35130700	2.79342600	-3.43071800
H	1.30867400	2.59341000	-3.92482700
H	0.03796100	1.65960300	-4.69549200
N	0.97772900	-1.11213100	-0.91889800
Pt	0.65979100	0.54243200	-2.36976600
N	-0.76578500	1.40787400	-1.13796500
H	-0.53038000	2.36590300	-0.96209700
H	-1.66946400	1.40935500	-1.57095000
H	-0.87740500	0.97643100	-0.23048700
Au	-0.46795800	-0.03208900	2.07312500
C	1.48370800	-0.84436100	2.41579600
H	2.28941000	-0.32221200	1.89312700
H	1.71765000	-0.76829700	3.47623900
H	1.57097100	-1.90278300	2.16035100
N	2.11959700	-0.26589400	-3.66504300
H	2.04748500	0.17568400	-4.56110400
H	3.04447800	-0.09129800	-3.32002300
H	2.02795900	-1.25326300	-3.80654600
C	-2.41939700	0.77754300	1.73948400
H	-2.97255500	0.30564800	0.92359800
H	-3.02078500	0.62674500	2.63430300
H	-2.42576100	1.85271200	1.54780700

Model 4 (MP2/def2-TZVP optimization)

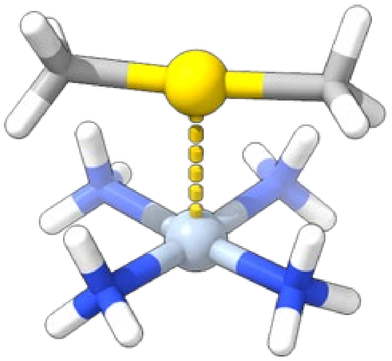

H	2.23892100	-1.49076800	-1.93463600
H	1.23686800	-1.78872900	-0.67473300
H	0.92159700	-2.40735300	-2.18815000
N	1.25606100	-1.58739400	-1.68173500
Pt	0.14394300	0.09594800	-1.90152400
N	-1.49289500	-0.96959700	-1.34986800
H	-2.38981300	-0.58016500	-1.63788200
H	-1.50892800	-1.93649300	-1.67364800
H	-1.46500700	-0.98886400	-0.32033100
Au	0.60796700	0.60574100	0.89254200
C	-0.13630500	-1.23656000	1.45831800
H	-1.21879600	-1.21321500	1.65909700
H	0.02788900	-2.11906700	0.81726100
H	0.31376300	-1.51145700	2.41767500
C	1.38543700	2.48451600	0.52667400
H	0.97865400	3.10051900	-0.29296000
H	1.24344000	3.09785600	1.42230000
H	2.47583700	2.46986400	0.37351700
N	1.80752100	1.19087200	-2.29216600
H	2.10231700	1.56465400	-1.37866800
H	2.58987600	0.67557600	-2.69398800
N	-0.93769200	1.81277900	-1.93777100
H	-1.94310600	1.69137400	-1.82067900
H	-0.61570500	2.34675900	-1.12159300
H	-0.82602000	2.38798100	-2.77283200
H	1.66611500	1.98479800	-2.91639500

Model 4 (RHF/def2-TZVP optimization)

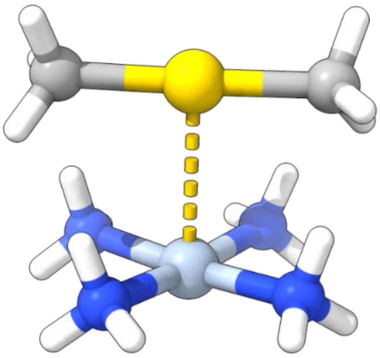

H	2.23923200	-1.57966800	-2.06956700
H	1.33880900	-1.81964600	-0.75238800
H	0.91974300	-2.47919600	-2.17851000
N	1.29349400	-1.65492700	-1.74596800
Pt	0.13122600	0.08410900	-1.97150600
N	-1.55226300	-1.03524400	-1.39585500
H	-2.43804000	-0.60698400	-1.58711600
H	-1.60610500	-1.95544800	-1.79098000
H	-1.47865500	-1.13750500	-0.39371900
Au	0.64274800	0.64047300	1.09121600
C	-0.16491200	-1.27620400	1.60684500
H	-1.25227900	-1.26623500	1.72032000
H	0.06694100	-2.13693200	0.96775400
H	0.21811500	-1.54967700	2.58568100
C	1.46065800	2.56830000	0.63695900
H	0.99055300	3.16987000	-0.15054200
H	1.39031200	3.18188800	1.53036800
H	2.52714600	2.53562100	0.39847000
N	1.84341600	1.23467300	-2.37532500
H	2.08338800	1.67793000	-1.50006000
H	2.64687300	0.71689900	-2.67691300
N	-0.99837500	1.85867500	-2.00145800
H	-1.99196300	1.73136900	-1.96435800
H	-0.73788900	2.35600200	-1.16408100
H	-0.82761500	2.46215800	-2.78430600
H	1.71737600	1.95927400	-3.05713600

Model S1 (MP2/def2-TZVP optimization)

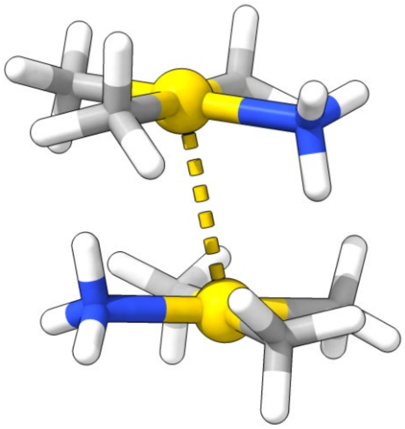

Au	0.00310000	1.68260000	-0.00910000
C	-0.83590000	1.85450000	1.90350000
C	-1.72540000	2.22790000	-0.88180000
C	0.83590000	1.62160000	-1.90760000
N	1.86790000	1.09480000	0.87070000
H	0.11270000	1.28480000	-2.65220000
H	1.17340000	2.62490000	-2.18850000
H	1.69500000	0.94390000	-1.94350000
H	2.04080000	1.54210000	1.76710000
H	2.65540000	1.28050000	0.25520000
H	-0.44510000	1.10790000	2.60480000
H	-0.56970000	2.84310000	2.29510000
H	-1.93000000	1.80580000	1.92880000
H	-2.17520000	1.33980000	-1.33210000
H	-2.40720000	2.66460000	-0.15260000
H	-1.51650000	2.94750000	-1.67360000
H	1.81290000	0.08660000	1.02750000
Au	-0.00310000	-1.68260000	-0.00910000
C	-0.83590000	-1.62160000	-1.90760000
C	1.72540000	-2.22790000	-0.88180000
C	0.83590000	-1.85450000	1.90350000
N	-1.86790000	-1.09480000	0.87070000
H	-1.17340000	-2.62490000	-2.18850000
H	-1.69500000	-0.94390000	-1.94350000
H	-0.11270000	-1.28480000	-2.65220000
H	-2.65540000	-1.28050000	0.25520000
H	-2.04080000	-1.54210000	1.76710000
H	1.93000000	-1.80580000	1.92880000
H	0.44510000	-1.10790000	2.60480000
H	0.56970000	-2.84310000	2.29510000
H	2.40720000	-2.66460000	-0.15260000
H	1.51650000	-2.94750000	-1.67360000

H	2.17520000	-1.33980000	-1.33210000
H	-1.81290000	-0.08660000	1.02750000
